# Physics-driven deep learning photoacoustic tomography

**DOI:** 10.1016/j.fmre.2024.06.014

**Published:** 2024-09-14

**Authors:** Kang Shen, Kuo Niu, Songde Liu, Yannis M. Paulus, Xiaohua Jiang, Chao Tian

**Affiliations:** a Department of Anesthesia, The First Affiliated Hospital of USTC, Division of Life Sciences and Medicine, University of Science and Technology of China, Hefei 230026, China; b Institute of Artificial Intelligence, Hefei Comprehensive National Science Center, School of Engineering Science, Hefei 230088, China; c School of Engineering Science, University of Science and Technology of China, Hefei 230026, China; d Department of Ophthalmology and Visual Sciences, University of Michigan, Ann Arbor 48105, USA; e Department of Biomedical Engineering, University of Michigan, Ann Arbor 48105, USA; f Center for Reproduction and Genetics, Department of Obstetrics and Gynecology, the First Affiliated Hospital of USTC, Division of Life Sciences and Medicine, University of Science and Technology of China, Hefei 230026, China; g Anhui Province Key Laboratory of Biomedical Imaging and Intelligent Processing, Institute of Artificial Intelligence, Hefei Comprehensive National Science Center, Hefei 230088, China

**Keywords:** Photoacoustic tomography, Image reconstruction, Deep learning, Filtered back projection, Incomplete projections

## Abstract

Photoacoustic tomography (PAT) is a rapidly emerging biomedical imaging modality. To achieve high-performance imaging, signal acquisition in PAT should ideally meet conditions such as full-view detection and dense sampling. However, these circumstances are rarely met in reality, leading to the ill-posed problem of image reconstruction from incomplete projections. Here we propose a physics-driven deep learning-based filtered back projection (dFBP) framework to address this important challenge. The dFBP network is inspired and constructed based on the physical model of the analytical filtered back projection algorithm and consists of a filtering module, a back-projection module, and a fusion module connected in cascade. The dFBP network is driven by physics and is thus interpretable and easy to train while being highly robust and versatile. Numerical and experimental validation on animals and humans show that dFBP-based PAT can achieve direct signal-to-image transformation with enhanced accuracy and reconstruct high-quality, artifact-suppressed images from sparse-view, limited-view, and acoustic heterogeneity-contaminated projections. The proposed dFBP provides a practical solution for high-performance PAT imaging under non-ideal conditions.

## Graphical abstract

**Figure:**
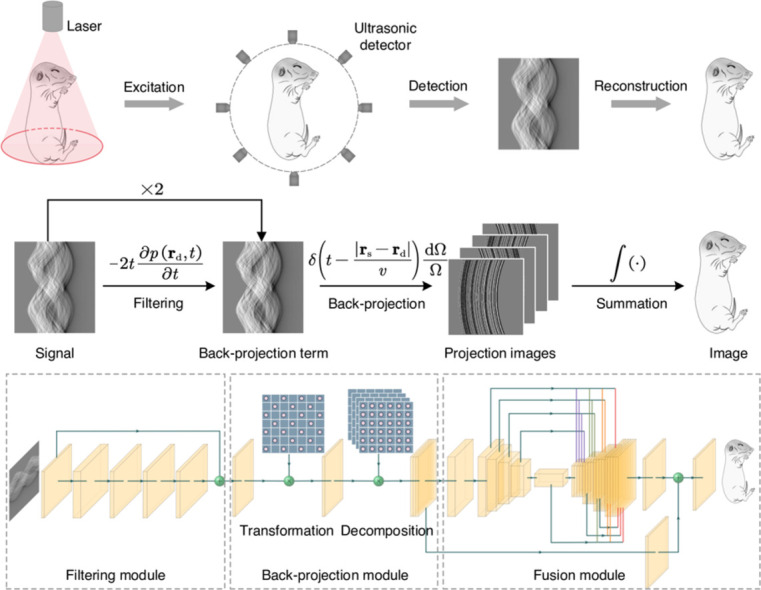

## 1 Introduction

Photoacoustic tomography (PAT) is a rapidly emerging biomedical imaging modality that has drawn considerable attention in the past two decades [[1], [2], [3]]. High-performance imaging is constantly being pursued in PAT and is essential for its widespread applications in biomedicine. To achieve high-performance imaging, the signal acquisition and processing in PAT should meet some ideal conditions. For example, full-view detection geometry should be employed for photoacoustic (PA) signal detection to avoid the possible loss of useful signals because the physical process of PAT is fundamentally three-dimensional (3D) [2]. High-density detector arrays and high-speed multichannel data acquisition systems should be deployed to ensure sufficient spatiotemporal sampling of PA signals [2]. In addition, the biological tissues under study should be ideally homogeneous to avoid signal contamination and image distortion [4]. However, these requirements can rarely be met in reality. Full-view signal detection is nonrealistic because full-view detection geometry spatially interferes with the illumination laser and/or biological tissues under study. Dense-view signal detection, acquisition, and processing are challenging due to the huge cost involved in the fabrication of high-density detector arrays and high-speed multichannel data acquisition systems. In addition, biological tissues are typically heterogeneous rather than homogeneous, which may cause image artifacts and degrade image quality. The violation of the ideal signal acquisition conditions leads to an ill-posed image reconstruction problem, which is highly challenging in PAT and other tomographic imaging modalities.

State-of-the-art image reconstruction algorithms in PAT mainly fall into three groups: back projection-based reconstruction, time reversal-based reconstruction, and iterative reconstruction. Back projection-based reconstruction is a class of analytical approaches attempting to recover PA images by inverting the Radon transform [5]. One well-known method is the filtered back-projection (FBP) algorithm proposed by Xu and Wang in 2005 [5]. The FBP algorithm can achieve accurate PAT image reconstruction when complete PA projection data are available. However, if projection data are incomplete, for example, in sparse- or limited-view imaging scenarios, significant artifacts and distortions may occur in the final reconstructed images [6,7]. Time reversal-based reconstruction recovers PA images by running a numerical acoustic propagation model backwards in time, where measured PA signals are retransmitted into the imaging region in temporally reversed order [8]. Time reversal algorithms can couple the acoustic properties of the media, such as dispersion, absorption, and acoustic speed, into the model and thus can work well for acoustically heterogeneous media. However, similar to FBP, time reversal is insufficient for sparse- and limited-view imaging problems. Iterative reconstruction performs PA inversion by minimizing a regularized objection function and is expected to achieve enhanced image reconstruction with incomplete projection data [9,10]. However, building inverse mathematical models with sufficient accuracy and appropriate regularization is difficult in practice. An inaccurate model or inappropriate regularization may induce undesired image artifacts. Moreover, iterative approaches require huge memory and are computationally expensive, which further restricts their applications, especially in demanding scenarios, such as real-time imaging and 3D imaging.

Recently, inspired by the tremendous success of deep learning in relevant fields, such as computer vision, natural language processing, and biomedical engineering, there has been a growing trend in employing deep learning techniques for tomographic image reconstruction in CT [11], MRI [12], PAT [13], and other imaging modalities [14]. Different from conventional physics-based image reconstruction, deep learning-based reconstruction techniques are data-driven approaches that automatically learn the mapping function between the input and output from training data. Moreover, the deep learning method employs complex non-linear transformations, such as convolutional layers and activation functions, allowing it to capture complicated relationships between input and output. These characteristics make deep learning methods have better performance compared with conventional methods. In PAT, deep learning techniques have been used to solve some problems, such as detector bandwidth expansion [15], resolution enhancement [16], low-energy imaging [17], image segmentation [18], noise removal [19], and sparse- and limited-view reconstructions [[20], [21], [22]]. In these studies, deep learning techniques primarily function as preprocessing methods in the data domain, postprocessing methods in the image domain, or enhancement modules for conventional algorithms. The fundamental image reconstruction process still highly depends on conventional image reconstruction methods. To achieve image reconstruction from raw PA projection data entirely relying on deep learning, a feature projection network (FPNet) [23] and an end-to-end UNet with residual blocks (Res-UNet) [24] were proposed, where the former employs a fully connected layer and the latter uses an end-to-end convolutional neural network. However, these methods require enormous amounts of training data to learn the complex mapping function between raw PA projection data and PA images and do not perform well for image reconstruction from incomplete data in experimental settings.

In this work, we propose a novel physics-driven deep neural network for high-quality image reconstruction from incomplete PA projections. The proposed approach possesses three distinct features. First, the architecture of the deep neural network is inspired and constructed based on the physical model of the analytical FBP algorithm; thus, we call the proposed approach deep FBP or dFBP. dFBP is robust and flexible because it inherits the robustness of FBP and the flexibility of deep learning. Second, the proposed dFBP goes significantly beyond the framework of state-of-the-art image reconstruction algorithms and can achieve direct 2D image reconstruction from 1D PA projection data with improved accuracy. Third, dFBP can achieve high-quality image reconstruction from sparse-view, limited-view, and acoustic heterogeneity-contaminated PA projection data and is well suited for PAT imaging under non-ideal conditions. The performance of dFBP is validated using numerical and *in vivo* datasets from animals to humans.

## 2 Methods

### 2.1 Forward model

In PAT, the generation and propagation of acoustic waves in a lossless homogeneous medium are governed by the wave equation

(1) $$\nabla^{2}p\left(r,t\right)-\frac{1}{v^{2}}\frac{\partial^{2}}{\partial t^{2}}p\left(r,t\right)=-\frac{\beta }{C_{p}}\frac{\partial }{\partial t}H\left(r,t\right),$$

where *p*(**r**, *t*) represents the acoustic pressure at position **r** and time *t, v* denotes the speed of sound, *β* is the isobaric thermal volume expansion coefficient, *C*_p_ is the specific heat capacity, and *H*(**r**, t) indicates the heating source. Applying Green's function approach to Eq. 1, the acoustic pressure (*i.e.*, PA signals) recorded by a detector at position **r**_d_ and time *t* can be written as [2]

(2) $$p\left(r_{d},t\right)=\frac{1}{4\pi v^{2}}\frac{\partial }{\partial t}\int_{V}\frac{p_{0}\left(r_{s}\right)}{\left|r_{s}-r_{d}\right|}\delta \left(t-\frac{\left|r_{s}-r_{d}\right|}{v}\right)dV,$$

where *p*_0_(**r**_s_) is the initial acoustic pressure at location **r**_s_ and d*V* is a 3D volume element. Fig. S1 in the Supplementary Materials shows the signal detection and image reconstruction geometry in PAT.

### 2.2 Analytical FBP

**Fig. 1:**
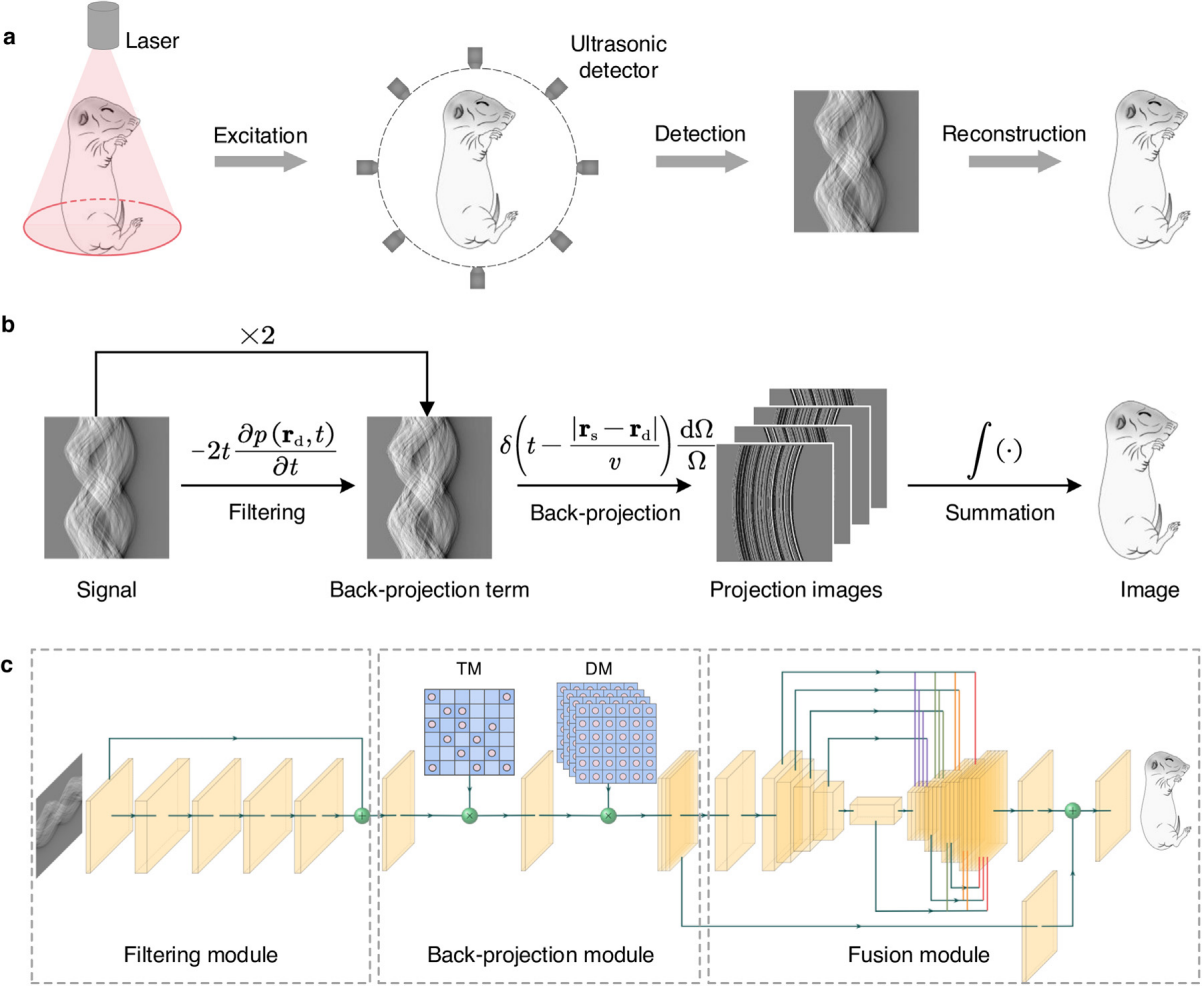
**Overview of the proposed dFBP approach and its network architecture.** (a) Schematic diagram showing the imaging principle of PAT. (b) Physical model of the analytical FBP-based image reconstruction. (c) Architecture of the proposed dFBP, which was inspired by the analytical FBP algorithm. The dFBP network consists of a filtering module, a back-projection module, and a fusion module. The operations involved in the network are as follows. Filtering module: 3 × Conv + a weighting matrix + a skip connection. Back-projection module: a sparse transformation matrix (TM) + a 3D decomposition matrix (DM). Fusion module: 1 × Conv + an encoder [4 × (2 × Conv + pooling) + 2 × Conv] + full-scale skip connections [(Conv + pooling/upsampling) for each connection line] + a decoder [4 × (Conv + upsampling) + 1 × Conv] + a skip connection for output (Conv).

The inversion problem in PAT is the process of image reconstruction (Fig. 1a), which aims to recover the initial acoustic pressure from measured PA projection data and can be mathematically modelled as

(3) $$p_{0}\left(r_{s}\right)=f\left(p(r_{d},t)\right),$$

where *f* is the transfer function of the image reconstruction problem. Different algorithms have different transfer functions and produce distinct reconstruction results. Back projection-based image reconstruction algorithms are a class of techniques that have analytical inversion formulas and achieve image reconstruction by inverting the spherical Radon transform in the forward model. Among them, one well-known technique is the filtered back-projection algorithm developed by Xu and Wang [5], where the reconstruction formula in Eq. 3 can be explicitly formulated as

(4) $$p_{0}\left(r_{s}\right)=\int_{\Omega }b\left(r_{d},t\right)\delta \left(t-\frac{\left|r_{s}-r_{d}\right|}{v}\right)\frac{d\Omega }{}.$$

The back-projection signal

(5) $$b\left(r_{d},t\right)=2\left[p\left(r_{d},t\right)-t\frac{\partial p(r_{d},t)}{\partial t}\right],$$

where Ω is the solid angle of the detection surface with respect to the reconstruction point and equals 2π for the infinite planar geometry and 4π for the spherical and cylindrical geometries. dΩ is the solid angle subtended by the element of the detection surface d*σ* and can be formulated as

(6) $$d\Omega =\frac{d\sigma }{{\left|r_{s}-r_{d}\right|}^{2}}\left(n_{d}\cdot \frac{r_{s}-r_{d}}{\left|r_{s}-r_{d}\right|}\right),$$

where **n**_d_ denotes the unit normal vector of the detection surface pointing to the region of interest (ROI). In FBP, the first derivative over time *t* is equivalent to PA signal filtering by a ramp filter in the frequency domain; the *δ* function indicates back projection; and the integration operation represents the summation process (Fig. 1b). Fig. S2 in the Supplementary Materials presents an example illustrating the reconstruction process of the analytical FBP algorithm.

### 2.3 dFBP network architecture

The dFBP network (Fig. 1c) was designed based on the reconstruction mechanism of the analytical FBP algorithm, which possesses three physical processes: signal filtering, back projection, and image summation. Mapping these physical processes into deep neural networks, a physics-driven dFBP net can be constructed. The dFBP net consists of three interpretable cascaded modules: a filtering module, a back-projection module, and a fusion module. The filtering module performs PA signal filtering and yields a back-projection signal. The subsequent back-projection module transforms the back-projection signal in the data domain into multiple intermediate projection images in the image domain. The fusion module integrates these projection images to form final PA images. The resultant transfer function of the dFBP network can be expressed as

(7) $$p_{0}\left(r_{s}\right)=f_{\text{fusion}}\left(f_{\text{back-projection}}\left(f_{\text{filtering}}\left(p\left(r_{d},t\right)\right)\right)\right),$$

where *f*_filtering_, *f*_back-projection_, and *f*_fusion_ represent the filtering, the back projection, and the fusion functions, respectively. Apparently, the input of the dFBP network is 1D raw PA signals yet the output is 2D PA images, indicating that dFBP can achieve direct signal-to-image transformation, which is distinct from state-of-the-art deep learning-based techniques.

#### 2.3.1 Filtering module

The filtering module consists of three convolutional layers, a matrix multiplication layer, and a skip connection operation (see Fig. 1c and Table S1 in the Supplementary Materials). The first convolutional layer contains 16 convolution kernels with a size of 1 × *L* × 1 (*L*: kernel length), followed by a Tanh activation function. The second convolutional layer contains 1 convolution kernel with a size of 1 × *L* × 16, followed by a Tanh activation function and a transpose operation. The last convolutional layer has *N*_s_ convolution kernels with a size of *N*_d_ × 1 × 1 (*N*_d_: number of detectors, *N*_s_: sampling points of temporal PA signals), followed by a transpose operation, where the Tanh activation function is not included. The three convolutional layers constitute the ramp filtering subnetwork in the filtering module (the ramp filtering subnetwork may also have other implementations, as discussed in Note S1 in the Supplementary Materials). The matrix multiplication layer is a matrix with dimensions of *N*_d_ × *N*_s_ × 1 that needs to be trained. It is used to compensate for the amplitude decay of PA signals during forward propagation [see the second term in Eq. 5]. The skip connection operation links the input raw PA signals with the output of the multiplication layer to form the back-projection signal. Note that the length of the convolution kernel *L* in the convolutional layers should generally be twice the length of raw PA signals. However, since the filtering operation in FBP [the ramp filter in Eq. 5] is achieved through multiple convolutional layers with multiple kernels in the filtering subnetwork, the value of *L* can be decreased to improve computational efficiency. In this work, *L* was set to a quarter of the length of the input PA signals.

#### 2.3.2 Back-projection module

The back-projection module transforms the back-projection signals output by the filtering module in the data domain to a series of back-projected images in the image domain. It is composed of a transformation matrix (TM) with a size of *N_x_N_y_* × *N*_d_*N*_s_ × 1 (*N_x_* and *N_y_*: width and height of output images) and a decomposition matrix (DM) with a size of *N_x_* × *N_y_* × *N*_d_ (Fig. 1c and Table S1 in the Supplementary Materials). The TM transforms the back-projection signals of all detectors into a low-quality 2D PA image and functions as the back-projection matrix in the analytical FBP algorithm [25] or the inverse of the system matrix in iterative reconstruction (IR), another commonly-used algorithm for PAT image reconstruction [26]. The DM decomposes the low-quality 2D PA image into a series of 2D projection images, each corresponding to the PA signal of a detector (see Fig. S14 in Note S2). This decomposition process helps preserve raw information of each signal channel [27], which can be exploited in the subsequent fusion module. The TM contains *N_x_N_y_* × *N*_d_ nonzero weights and is essentially a sparse matrix. The DM consists of *N*_d_ 2D matrices with a size of *N_x_* × *N_y_* and can be obtained by reshaping the TM.

In the implementation, the input (back-projection signals) is first reshaped into a 1D data-domain vector with a size of *N*_d_*N*_s_ × 1 and transformed into a 1D image-domain vector by the TM. The 1D image-domain vector is then reshaped into a 2D image having a size of *N_x_* × *N_y_*. The 2D image is finally multiplied with the DM and is decomposed into *N*_d_ projection images, which serves as the input of the fusion module. Note that the data-to-image transformation in the back-projection module is achieved by a TM rather than a neural network, such as a fully connected layer described in Note S2 in the Supplementary Materials. This is because the use of a TM can dramatically reduce the number of parameters involved in network training and relax the requirements on the amount of training data and computational memory.

#### 2.3.3 Fusion module

The architecture of the fusion module (Fig. 1c and Table S1 in the Supplementary Materials) is constructed based on a full-scale connected U-Net [28]. The module mainly contains four parts: a convolutional layer, an encoder, a decoder, and a series of skip connections. The convolutional layer at the beginning has 64 kernels and is used to process the input data (the 3D projection matrix output by the back-projection module) to reduce possible information loss at the initial stage. The encoder is made up of four contractive blocks and two convolutional layers at the end. Each contractive block contains two convolutional layers and a downsampling layer (*i.e.*, a pooling layer). While the convolutional layers in the first block have 64 kernels, the convolutional layers in subsequent blocks have progressively doubled kernels along the contractive path. Both convolutional layers at the end have 1024 kernels and process the output features of the fourth contractive block to produce the output of the encoder. The decoder is made up of four expansive blocks and a convolutional layer at the end. Each expansive block includes one convolutional layer with 64 kernels and an upsampling layer. The convolutional layer at the end has one 3 × 3 kernel with no normalization or activation and processes the output features of the fourth expansive block to yield the output of the decoder. Complementary to the encoder that generates compressed features, the decoder restores the size of the compressed features to the original. To take full advantage of the features at different levels, full-scale skip connections containing a convolutional layer and a resizing layer are introduced into the fusion module (Table S1 in the Supplementary Materials). The convolutional layer similarly has 64 kernels. The resizing layer ensures image size matching and is either a downsampling layer or an upsampling layer according to the size of the features to be aggregated. The final output of the fusion module is the output of the decoder linked with the processed input data of the fusion module using a 1 × 1 convolution kernel with no normalization or activation (Fig. 1c). All convolution kernels involved above have a size of 3 × 3, followed by a group normalization operation and a ReLU activation function unless otherwise specified. Although batch normalization is the most widely used strategy for feature normalization, this work adopts group normalization because the latter performs better for small batches. See Note S3 in the Supplementary Materials for the descriptions of batch normalization and group normalization.

### 2.4 Datasets generation

To train and test the dFBP network, we constructed a numerical mouse embryo dataset, a numerical zebrafish dataset, an *in vivo* mouse dataset, and an *in vivo* human finger dataset (Table S2 and Note S4 in the Supplementary Materials). These datasets were separately used for the training of the dFBP network to ensure optimal reconstruction performance over certain tasks. This strategy also allows us to adjust the size of the network according to the size of the training data.

#### 2.4.1 Numerical mouse embryo dataset

The dataset contains 2878 image slices acquired from 51 mouse embryos by optical projection tomography [29]. The images were randomly divided into three subsets: a training set (80%), a validation set containing (10%), and a test set (10%). We also collected 10 hematoxylin and eosin (H&E)-stained histological images of a mouse embryo as additional samples to test the robustness of dFBP [30]. To facilitate training, all images were resized to 192 × 192 pixels. Raw PA signals were generated using the k-Wave toolbox [31], where the PA signal detection geometry is a closed sphere (diameter: 50 mm) with 2048 evenly distributed detectors.

#### 2.4.2 Numerical zebrafish dataset

The dataset consists of 1112 micro-computed tomography (micro-CT) images from 5 zebrafish [32] and was expanded to 4448 images through simple data augmentation. Among the 5 zebrafish, 4 zebrafish containing 3556 slices were used to train the dFBP network and the remaining one was equally split for validation (444 slices) and testing (448 slices). The dataset was employed in both the sparse-view and the limited-view PAT imaging. In sparse-view imaging, PA signals generated from the training set are collected by 32, 64, 128, 256, and 512 detectors evenly distributed over a circle with a diameter of 80 mm. In limited-view imaging, PA signals generated from the training set are collected by 80-mm-diameter, equally-spaced, partially or fully circular detector arrays with central angles of π/4, π/2, 3π/4, π, and 2π. To facilitate training, all zebrafish images were resized to 192 × 384 pixels. Raw PA signals were generated using the k-Wave toolbox [31].

#### 2.4.3 Experimental setup

The schematic of our custom-built full-ring detector array-based PAT imaging system is shown in Fig. S3 in the Supplementary Materials. The laser source used for illumination is provided by a wavelength-tunable (690–950 nm, 1200–2400 nm) optical parametric oscillator (OPO) laser (Phocus HE Core, Opotek Inc., CA, USA) with a repetition rate of 10 Hz and a pulse duration of 5 ns. The laser beam is first coupled into a fibre bundle and then split into 12 groups, which are evenly positioned around the sample under study and form a uniform ring-shaped illumination pattern. Generated PA signals are recorded by a full-ring transducer array (diameter: 80 mm, number of elements: 512, center frequency: 5 MHz, bandwidth: > 65%; Imasonic sas, France), whose elements are cylindrically focused in the elevational direction. The PA signals received by the transducer array are digitized by a 256-channel commercially-available data acquisition system (Vantage 256, Verasonics Inc., WA, USA) through time division multiplexing. The data acquisition system and the OPO laser are synchronized through a function generator. The developed PAT imaging system can be used for high-performance small animal whole-body imaging by scanning the animal or the transducer array along the elevational direction.

#### 2.4.4 *In vivo* mouse dataset

The animal experiments were performed in accordance with the National Institute of Health (NIH) Guide for the Care and Use of Laboratory Animals, after approval of the laboratory animal protocol by the Institutional Animal Care and Use Committee (IACUC) of the University of Science and Technology of China (Protocol Number USTCACUC1803065). The dataset was created by performing tomographic scanning of 15 living mice using the custom-built 512-channel full-view PAT imaging system (diameter: 80 mm) (Fig. S3 in the Supplementary Materials). Each mouse generates about 200 cross-sectional whole-body images. Among the 15 mice, 12 mice were used for the training of the dFBP network, 2 mice were used for validation, and 1 mouse was used for testing. The dataset was employed in both the sparse-view and the limited-view PAT imaging in this study. In sparse-view imaging, 512-channel raw PA projection data were downsampled by factors of 16, 8, 4, and 2 to yield 32-, 64-, 128-, and 256-channel experimental projection data, respectively. In limited-view imaging, PA projection data with a view angle of π/4, π/2, 3π/4, and π were generated by consecutively extracting 64-, 128-, 192-, and 256- channel data from 512-channel full-view projection data, respectively.

#### 2.4.5 *In vivo* human finger dataset

The studies on human subjects have been approved by the Institutional Review Board (IRB) of the First Affiliated Hospital of the University of Science and Technology of China (IRB: 2022-ky357) and adhered to the tenets of the Declaration of Helsinki. All subjects were provided with written informed consent prior to the study. The human finger dataset contains 963 cross-sectional images generated from 10 index or middle fingers of 5 male volunteers using the developed full-view PAT imaging system. The data are randomly divided into three sets: a training set containing 768 images from 8 fingers, a validation set containing 97 images from 1 finger, and a test set containing 98 images from 1 finger. In limited-view imaging, PA projection data with view angles of π/2, π, and 3π/2 were generated by consecutively extracting 128-, 256-, and 384-channel data from 512-channel full-view projection data, respectively.

### 2.5 Training strategies

The proposed dFBP network has three modules in cascade. The training strategy adopted in this work is balanced module-by-module training and has three stages. Specifically, in the first stage, we only trained the filtering module. The input is raw PA projection data and the output is the reference back-projection data theoretically generated using Eq. 5. In the second stage, we trained the filtering module and the TM in the back-projection module simultaneously, where the TM is the focus and the filtering module was fine-tuned. The input is raw PA projection data and the output is the PA images reconstructed by the analytical FBP method using the input raw PA projection data. The DM is subsequently obtained by reshaping the TM (see Section 2.3.2). In the third stage, we trained the dFBP network as a whole, where the fusion module is the focus and the filtering and the back-projection modules were fine-tuned. The input is still the raw PA projection data and the output is the PA images reconstructed by the analytical FBP algorithm using reference projection data (*i.e.* projection data acquired in dense- and full-view imaging).

The first and second stages of the training process estimate the approximate weights of the filtering and back-projection networks, and the third stage of the training process refines their values and determines the weights of the fusion network. Principally, the dFBP network can be trained in a single end-to-end process. However, this may result in instability due to the complexity of the network. The module-by-module training strategy adopted in this study can estimate optimal weights for each module, thereby enhancing the overall stability of the dFBP network. The batch sizes were set to 1, 16, and 3 for the three training stages. The mean-square loss function was used as the objective function, and the Adam optimizer was employed to seek optimal network weights with a learning rate of 1.0 × 10^–4^ for the first and third stages and 2.5 × 10^–5^ for the second stage. All training processes were implemented under the TensorFlow 2.0 framework and the codes were deployed on a single NVIDIA RTX TITAN GPU for accelerated computation.

### 2.6 Statistics

We performed a power analysis using a two-sided *t*-test. We anticipated that the dFBP algorithm would result in at least a 50% improvement in RMSE values. Power calculations were performed so that numbers allow detection of statistically significant differences between treatment groups with a power of 0.90, α of 0.05, and an anticipated large effect size (Cohen's d value) of 1.0. This calculation determines a minimum of 13 animals needed. Considering possible sample loss due to animal mortality and exclusion, 15 animals were used in this study. A *p* < 0.05 is deemed statistical significance.

## 3 Results

### 3.1 dFBP achieves direct signal-to-image transformation and outperforms analytical FBP in terms of accuracy

**Fig. 2:**
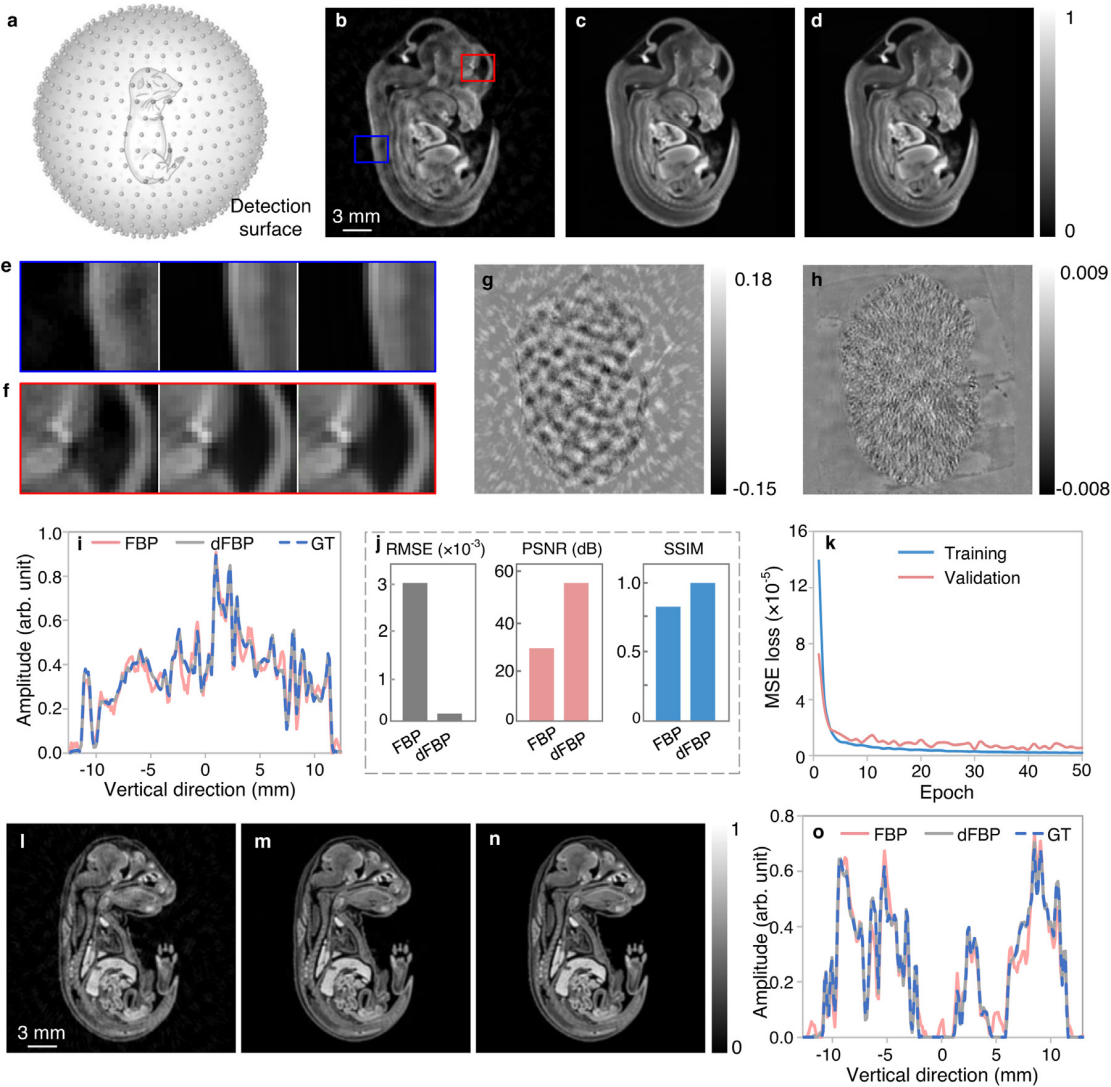
**dFBP achieves robust image reconstruction and outperforms analytical FBP in accuracy.** (a) Schematic diagram showing the configuration of the simulation. The PA signals generated from a numerical mouse embryo are recorded by 2048 point detectors evenly distributed over a spherical measurement surface (diameter: 50 mm). (b)-(d) Reconstruction results of a representative slice from the test dataset. (b) Image reconstructed by FBP. (c) Image reconstructed by dFBP. (d) Reference image. (e) and (f) Close-up views of the regions within the blue and red boxes in (b)-(d). (g) Difference image between the FBP-reconstructed image and the reference image. (h) Difference image between the dFBP-reconstructed image and the reference image. (i) Comparison of the intensity profiles along the vertical line in the middle of the images in (b)-(d). (j) Quantitative evaluation of the results reconstructed by FBP and dFBP on the test dataset. (k) Loss curves on the training dataset and the validation dataset. (l)-(n) Reconstruction results of a representative H&E-stained histology slice from the other test dataset. (l) Image reconstructed by FBP. (m) Image reconstructed by dFBP. (n) Reference image. (o) Comparison of the intensity profiles along the vertical line in the middle of the images in (l)-(n). GT: ground truth.

To assess the performance of the proposed approach, the numerical mouse embryo dataset is used to train the dFBP network. After training, the dFBP network was evaluated on an independent test dataset. The PA signal detection geometry employed in the simulation is schematically shown in Fig. 2a, where the PA signals generated from the mouse embryo are collected by 2048 point-like ultrasonic detectors evenly distributed over a sphere with a diameter of 50 mm.

The imaging results of a representative slice of the mouse embryo by FBP and dFBP are shown in Fig. 2b and c, respectively, and the corresponding reference image is displayed in Fig. 2d. For comparison, the close-up views of the regions within the blue and red boxes in Fig. 2b-d are depicted in Fig. 2e, f. The results show that analytical FBP achieves good reconstruction, but the result possesses obvious visual differences from the reference image. Benefitting from the high adaptability and excellent feature-processing capability, dFBP reproduces the reference image with high accuracy and substantially improves the image quality. The difference images (Fig. 2g,h) and the comparison of the intensity profiles of a vertical line (Fig. 2i) corroborate the superior performance of dFBP. The quantitative analysis of the reconstruction results on the entire test dataset was further conducted based on three metrics, including RMSE (root-mean-square error), PSNR (peak signal-to-noise ratio), and SSIM (structure similarity index measure) (Note S5 in the Supplementary Materials). These quantitative measures (Fig. 2j) show that dFBP yields images with lower RMSE and higher PSNR and SSIM values. Additionally, the training- and validation-loss curves (Fig. 2k) indicate that although the network is trained to fit the training dataset, it also works well on the validation dataset, which again confirms the feasibility of the proposed approach. To evaluate the robustness of dFBP, another independent test dataset containing 10 hematoxylin and eosin (H&E)-stained histological images of a mouse embryo visually different from the training OPT images was also used for testing. The imaging results (Fig. 2l-n) and the intensity profiles of a vertical line (Fig. 2o) demonstrate that although the dFBP network is not trained using the histological images, it similarly achieves high-quality image reconstruction and outperforms analytical FBP in terms of accuracy (Fig. 2o). In addition to the simulations, an experimental study on the feasibility of dFBP-based PAT image reconstruction is performed in parallel and shown in Fig. S4 in the Supplementary Materials.

### 3.2 dFBP-based PAT achieves high-quality imaging under sparse-view measurements

To achieve accurate imaging, most image reconstruction algorithms require that PA signals from biological tissues be sampled in dense views. In practice, however, detectors for PA signal acquisition are usually sparsely arranged due to high fabrication costs and the requirement for real-time imaging, which results in the problem of sparse-view imaging. Image reconstruction from sparse projection data is mathematically ill-posed, and conventional algorithms such as FBP fail to yield satisfactory results, especially when the number of projections is small. Here we numerically and experimentally demonstrate that dFBP can achieve high-quality image reconstruction from sparse-view projection data.

**Fig. 3:**
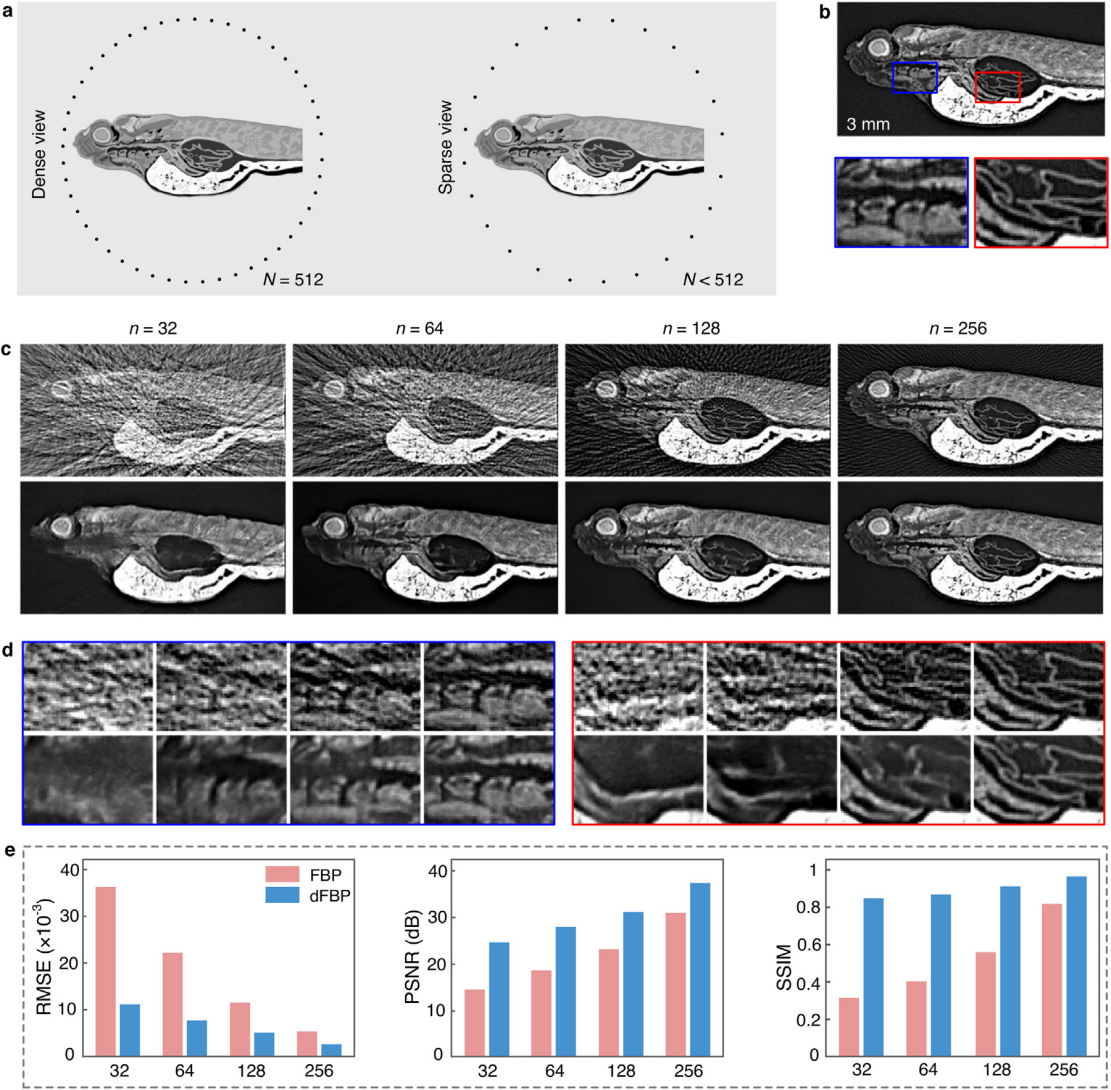
**dFBP-based PAT achieves high-quality imaging under sparse-view measurements.** (a) Schematic diagrams showing the configuration of the zebrafish imaging simulation under dense-view and sparse-view measurements. (b) Reference image of a numerical zebrafish and close-up views of the regions within the blue and red boxes. (c) Imaging results. The first and second rows are the images reconstructed by FBP and dFBP under different numbers of views (from left to right: *n* = 32, 64, 128, and 256). (d) Close-up views of the regions within the blue and red boxes under different numbers of views. The first and second rows are the reconstruction results of FBP and dFBP, respectively. (e) Quantitative evaluation of the results on the test dataset. Left: RMSE; middle: PSNR; right: SSIM. For comparison, the contrast of all reconstructed images in this example and this study was adjusted equally.

We first numerically evaluated the performance of dFBP on the zebrafish dataset. The signal detection geometry in this case is schematically shown in Fig. 3a, where the PA signals generated from the zebrafish slices are collected by 32, 64, 128, 256, and 512 point-like detectors evenly distributed over a circle with a diameter of 80 mm. The images reconstructed by FBP using the projection data of 512 detectors are used as the reference images for network training. After training, the dFBP network was evaluated on the test dataset. Fig. 3c shows the reconstruction results of a representative image (Fig. 3b) with high dynamic frequency and brightness ranges by FBP and dFBP from 32-, 64-, 128-, and 256-channel projection data. Fig. 3d presents close-up views of the reconstruction results within the blue and red boxes (Fig. 3b). As expected, visible artifacts are present in the images reconstructed by FBP, especially when the number of detectors is small. In contrast, dFBP suppresses artifacts, recovers more structures, and produces images with enhanced quality for all cases. To further evaluate the performance of the method, the quantitative analysis of the imaging results was conducted (Fig. 3e). The results show that dFBP has substantially better performance than the analytical FBP algorithm in terms of RMSE, PSNR, and SSIM and is preferable in sparse-view PAT imaging. For more information, Fig. S5 in the Supplementary Materials provides the difference images between the reference image (Fig. 2b) and the reconstructed images (Fig. 3c) and Fig. S6 in the Supplementary Materials showcases the imaging results of other zebrafish slices.

**Fig. 4:**
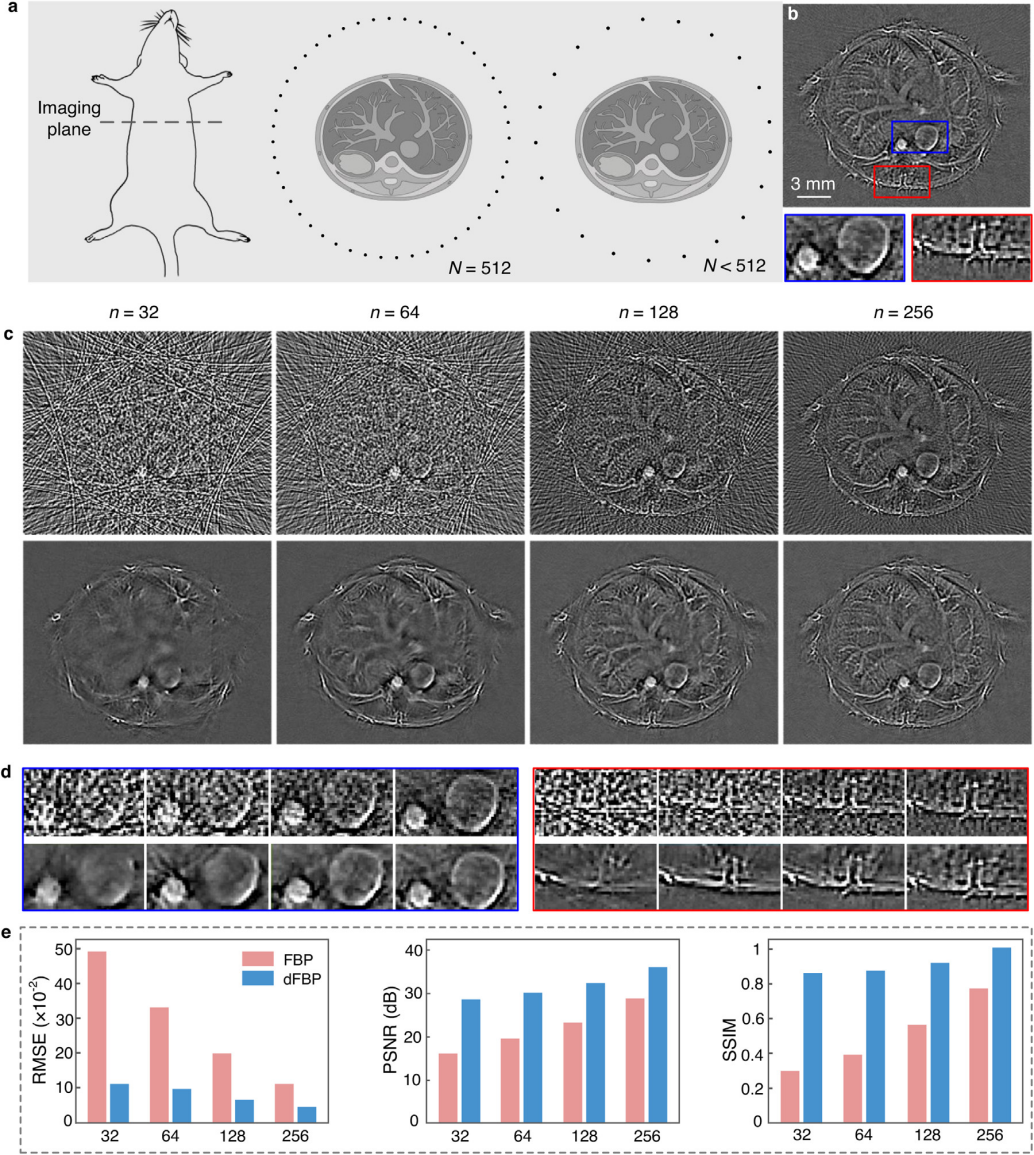
**dFBP-based PAT achieves high-quality *in vivo* imaging under sparse-view measurements.** (a) Schematic diagram showing the configuration of the *in vivo* mouse imaging experiment under dense-view and sparse-view measurements. (b) Reference image of a transverse section of a mouse at the level of the liver and close-up views of the regions within the blue and red boxes. (c) Imaging results. The first and second rows are the images reconstructed by FBP and dFBP under different numbers of views (from left to right: *n* = 32, 64, 128, and 256). (d) Close-up views of the regions within the blue and red boxes under different numbers of views. The first and second rows are the reconstruction results of FBP and dFBP, respectively. (e) Quantitative evaluation of the reconstruction results on the test dataset.

We then experimentally evaluated the performance of dFBP on the *in vivo* mouse dataset. Fig. 4a shows the experimental signal detection geometry, where the PA signals generated from the living mice are collected by 32, 64, 128, 256, and 512 detectors evenly distributed over the full-ring detector array. Similar to the numerical zebrafish dataset, the images reconstructed by FBP using the projection data of 512 detectors are used as the reference images for network training. After training, the dFBP network was evaluated on the test dataset. Fig. 4c shows a representative tomographic image (Fig. 4b) reconstructed by FBP and dFBP from 32-, 64-, 128-, and 256-channel projection data. Fig. 4d presents close-up views of the reconstruction results within the blue and red boxes (Fig. 4b). Similarly, the experimental mouse images reconstructed by FBP suffer from significant streak artifacts and are of inferior image quality. This is especially true when the number of detectors is less than 256. In contrast, dFBP successfully reconstructs major vessel structures even using highly downsampled projection data and possesses superior image quality. It is worth noting that although dFBP fails to produce high-quality images when the number of detectors is extremely small, for example, 32, the resultant image still possesses better quality than that of FBP. The quantitative analysis of the results shows that dFBP outperforms FBP on the experimental dataset in terms of RMSE, PSNR, and SSIM (Fig. 4e), consistent with the conclusion drawn in the numerical simulations. For more information, Fig. S7 in the Supplementary Materials provides the difference images between the reference image (Fig. 4b) and the reconstructed images (Fig. 4c). For practical applications of the dFBP algorithm in sparse-view imaging, it is recommended that a minimum of 64 detectors should be used to yield images with acceptable image quality.

### 3.3 dFBP-based PAT achieves high-quality imaging under limited-view measurements

The view angle (Ω) of a PA signal detection geometry is critical to the image quality in PAT. To obtain high-quality images, PA sources should be enclosed by the measurement geometry as much as possible [2]. Ideally, the view angle should be 4π steradians (Ω = 4π steradians) for 3D imaging or 2π radians (Ω = 2π radians) for 2D imaging. However, the view angle of the measurement geometry is usually limited in practical scenarios, making it impossible to collect useful signals from all directions and resulting in the problem of limited-view imaging. Image reconstruction from limited-view projections is also mathematically ill-posed, and conventional algorithms such as FBP fail, especially when the view angle is small. Here we demonstrate that dFBP can recover high-quality PA images from limited-view projection data in both numerical simulations and *in vivo* experiments.

**Fig. 5:**
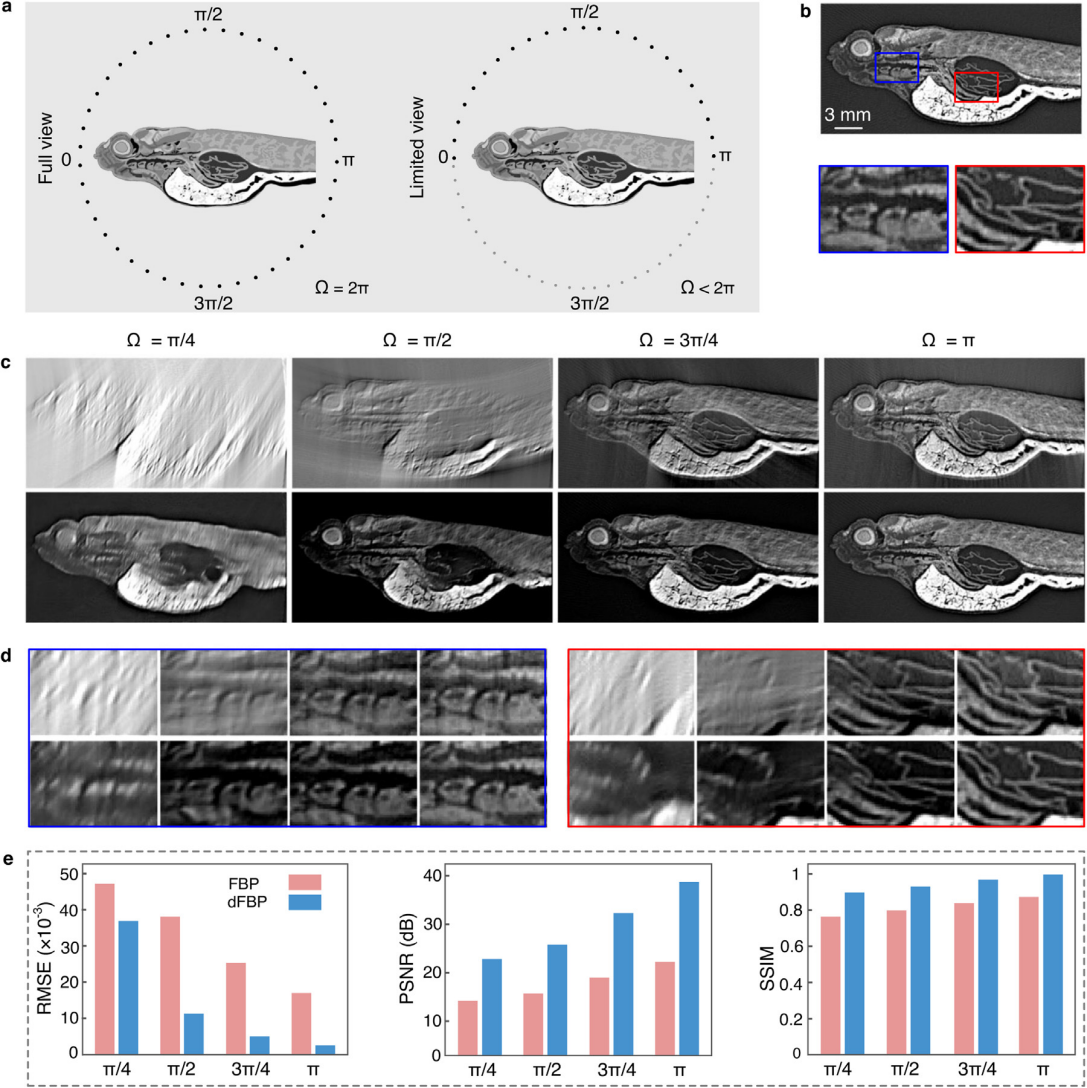
**dFBP-based PAT achieves high-quality imaging under limited-view measurements.** (a) Schematic diagram showing the configuration of the zebrafish imaging simulation under full-view and limited-view measurements. (b) Reference image of a numerical zebrafish and close-up views of the regions within the blue and red boxes. (c) Imaging results. The first and second rows are the images reconstructed by FBP and dFBP under different view angles (from left to right: Ω *=* π/4, π/2, 3π/4, and π). (d) Close-up views of the regions within the blue and red boxes. The first and second rows are the reconstruction results of FBP and dFBP, respectively. (e) Quantitative evaluation of the results on the test dataset.

The numerical simulations were carried out based on the zebrafish dataset. The signal detection geometry in this case is shown in Fig. 5a, where the PA signals generated from the zebrafish are collected by 512 detectors evenly distributed over a full-view (Ω = 2π) circular detector array with a diameter of 80 mm. To simulate the limited-view imaging scenarios, the shape of the detector array reduces from a full circle to partial circles with central angles of π/4, π/2, 3π/4, and π. The images reconstructed by analytical FBP with full-view projection data are used as the reference images for network training. After training, the dFBP network was evaluated on the test dataset. Fig. 5c shows the reconstruction results of a representative image (Fig. 5b) by FBP and dFBP under view angles of π/4, π/2, 3π/4, and π. Fig. 5d presents the close-up views of the reconstruction results within the blue and red boxes (Fig. 5b). Unsurprisingly, significant distortions, blurring, and artifacts occur in the images reconstructed by FBP due to the limited detection angles. This is especially true when the view angle is small. In contrast, dFBP suppresses artifacts, recovers fine structures, and produces images with enhanced quality. The quantitative analysis of the results (Fig. 5e) demonstrates that dFBP outperforms FBP for all cases in terms of RMSE, PSNR, and SSIM and is a preferable algorithm for limited-view PAT imaging. For more information, Fig. S8 in the Supplementary Materials provides the difference images between the reference image (Fig. 5b) and the reconstructed images (Fig. 5c), and Fig. S9 in the Supplementary Materials showcases the imaging results of other cross-sectional slices.

**Fig. 6:**
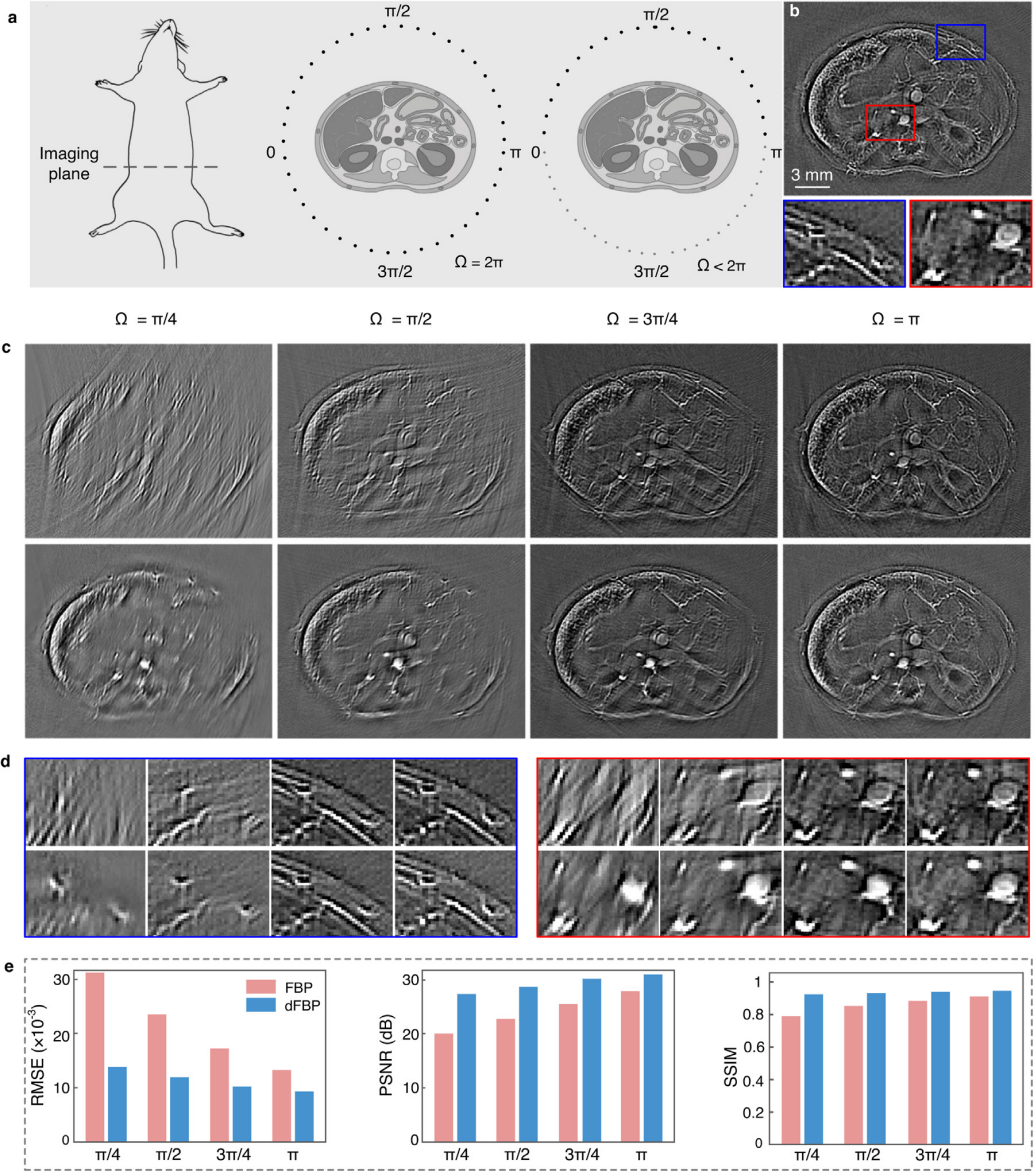
**dFBP-based PAT achieves high-quality *in vivo* imaging under limited-view measurements.** (a) Schematic diagrams showing the configuration of the *in vivo* mouse imaging experiment under full-view and limited-view measurements. (b) Reference image of a transverse section of a mouse at the level of the kidney and close-up views of the regions within the blue and red boxes. (c) Imaging results. The first and second rows are the images reconstructed by FBP and dFBP under different view angles (from left to right: Ω *=* π/4, π/2, 3π/4, and π). (d) Close-up views of the regions within the blue and red boxes. The first and second rows are the reconstruction results of FBP and dFBP, respectively. (e) Quantitative evaluation of the results on the test dataset.

The experimental study was conducted based on the *in vivo* mouse dataset. The experimental signal detection geometry (Fig. 6a) in this case is similar to the configuration in the simulation study except that the imaging target is living mice instead of numerical zebrafish. The shape of the detector array similarly reduces from a full circle to partial circles with central angles of π/4, π/2, 3π/4, and π. The images reconstructed by analytical FBP with full-view projection data are used as the reference images for network training. Fig. 6c shows the reconstruction results of a representative image (Fig. 6b) in the test dataset by FBP and dFBP under view angles of π/4, π/2, 3π/4, and π. Fig. 6d presents close-up views of the reconstruction results within the blue and red boxes (Fig. 6b). The results again show that FBP produces images with distortions and artifacts, especially when the view angle of the detection geometry is small, while dFBP reduces distortion, suppresses artifacts, and yields images with enhanced quality. The quantitative metrics of the experimental results (Fig. 6e) similarly demonstrate the superiority of dFBP over FBP. For more information, Fig. S10 in the Supplementary Materials presents the difference images between the reference image (Fig. 6b) and the reconstructed images (Fig. 6c). For practical applications of the dFBP algorithm in limited-view imaging, it is recommended that detectors with a minimum of π/2 view angle should be used to yield images with acceptable image quality.

### 3.4 dFBP-based PAT achieves high-quality imaging under limited-view measurements with acoustic heterogeneity

Image formation in PAT is generally based on the assumption that biological tissues are acoustically homogeneous. However, this does not hold, especially when strongly heterogeneous tissues, such as bones and air cavities, are present. Tissue heterogeneity can cause acoustic reflection, refraction, and scattering at interfaces, which may induce distortions and artifacts in final images [4,33]. One simple and effective approach for correcting acoustic heterogeneity-induced image artifacts is the half-time reconstruction method proposed by Anastasio and colleagues [34], which simply discards contaminated projection data in the time domain. While the half-time approach works well for full-view PAT imaging, it fails to yield images with reasonable quality in limited-view imaging scenarios (see Fig. S11 in the Supplementary Materials). Here we demonstrate that the proposed dFBP method can effectively address the acoustic heterogeneity problem and produce enhanced reconstruction results even in limited-view PAT imaging.

**Fig. 7:**
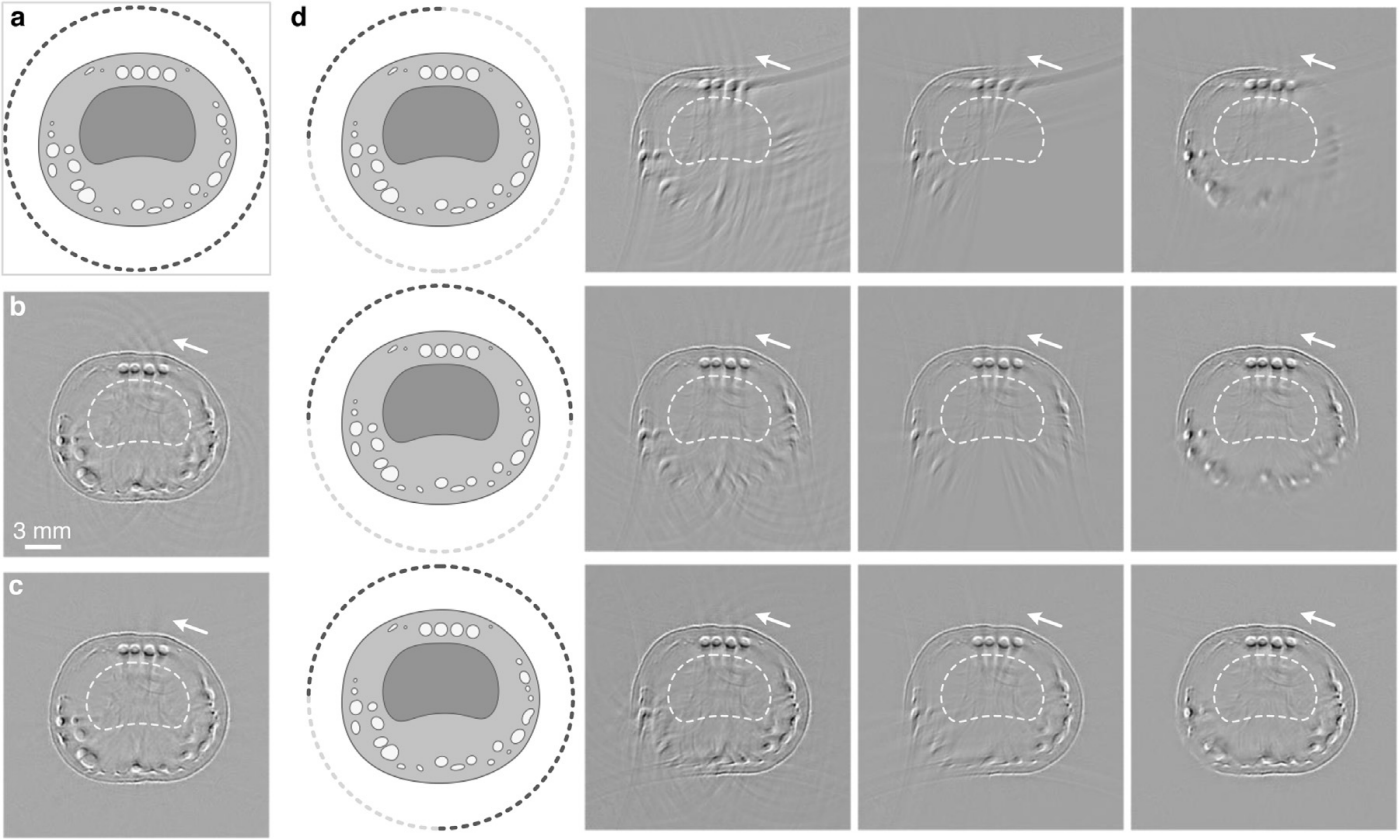
**dFBP-based PAT achieves high-quality imaging of human fingers under limited-view measurements with acoustic heterogeneity.** (a) Schematic diagram showing the configuration of the finger imaging experiment using a circular detector array. Finger bone (phalanx) is a strong acoustically heterogeneous medium that contaminates PA signals and induces image artifacts. (b) Image reconstructed by FBP using full-time full-view (Ω = 2π) projection data. Artifacts are prominent. (c) Image reconstructed by FBP using half-time full-view (Ω = 2π) projection data. Artifacts are less significant. The images are used as reference images for the training of the dFBP network. (d) Images reconstructed by FBP and dFBP using full-time and half-time projection data at different view angles. First row to third row: Ω = π/2, π, and 3π/2. First column to third column: FBP reconstruction with full-time projection data, FBP reconstruction with half-time projection data, and dFBP reconstruction with full-time projection data. The white dashed lines represent the boundary of the phalanx of the finger.

The demonstration proceeds with a human finger imaging example (Fig. 7a), where phalanxes in fingers can be regarded as strong acoustically heterogeneous media. The finger data used for the network training are paired, indicating that the input of the network is full-time limited-view projection data and the output is the reference images reconstructed by the analytical FBP algorithm using half-time full-view projection data. Fig. 7b,c presents the images reconstructed by FBP using full-time full-view projection data and half-time full-view projection data, respectively. As expected, the image reconstructed using full-time projection data suffers from image artifacts due to signal contamination by finger bones [4] while the image reconstructed using half-time projection data is less affected and used as the reference for network training. After training, the dFBP network was evaluated on the test dataset. Fig. 7d showcases the reconstruction results of a representative test image by FBP and dFBP using full-time and half-time projection data when the view angles are π/2, π, and 3π/2. In the limited-view imaging cases, the images reconstructed by FBP with full-time projection data contain prominent distortions and artifacts, especially when the view angle is small. The images reconstructed by FBP with half-time projection data lose a substantial portion of image structures and have even worse quality. In contrast, dFBP mitigates image distortions, suppresses reflection artifacts (see white arrows), and substantially improves image quality. These results demonstrate the superior capability of dFBP in correcting acoustic heterogeneity-caused image degradation in limited-view PAT imaging. It is worth noting that under the same view angle, for example, Ω = π, the images reconstructed by FBP and dFBP in this example possess lower quality than those displayed in the *in vivo* mouse imaging experiment (Fig. 6c). This is because finger bones in this example hinder the normal propagation of PA signals and worsen the limited-view imaging problem.

## 4 Discussion and conclusion

The difficulty of network training is an important measure for evaluating the performance of a deep neural network. Learning direct mapping functions from PA signals to PA images is difficult, especially when projection data are incomplete. Using a general convolutional neural network or a fully connected layer to approximate the mapping function requires huge training data and is computationally intensive [35]. The proposed dFBP network incorporates the physical model of the analytical FBP algorithm and learns direct signal-to-image mapping in a much easier and more effective manner. Note S6 in the Supplementary Materials demonstrates that the practical performance of dFBP is substantially superior to state-of-the-art deep learning-based PAT image reconstruction algorithms, such as Res-UNet [24] and FPNet-UNet [23], which employ a fully connected layer and an end-to-end convolutional neural network for image reconstruction, respectively. In addition to the physics-driven design of the network architecture, another key feature in constructing the dFBP network is that the transformation of filtered PA signals in the data domain to back-projected PA images in the image domain is realized based on a 2D sparse matrix and a small-scale 3D matrix (Fig. 1c) rather than a fully connected layer adopted in some algorithms. This reduces the number of parameters to be trained by three orders of magnitude (from *N_x_N_y_* × *N*_d_*N*_s_ to *N_x_N_y_* × *N*_d_*, N_x_* and *N_y_*: width and height of output images, *N*_d_: number of detectors, *N*_s_: sampling points of temporal PA signals) and allows the network to be trained using less data and less computation.

Robustness and versatility are other important considerations for a deep neural network. The dFBP network is driven by the physical model of the analytical FBP algorithm and is demonstrated to be highly robust. It can cope with different imaging scenarios and yield high-quality images across a range of samples from animals to humans, including mouse embryos, zebrafish, living mice, and human fingers. In addition, dFBP inherits the versatility of analytical FBP and poses no requirements on the shape of detection geometry and the size of input signals.

In conclusion, we propose a novel physics-driven deep filtered back-projection framework called dFBP to address the challenging ill-posed image reconstruction problem in PAT under nonideal conditions. The dFBP network is constructed based on the physical model of the analytical FBP algorithm and is thus interpretable. It consists of a filtering module, a back-projection module, and a fusion model corresponding to the filtering, back projection, and summation operations in analytical FBP and can achieve direct signal-to-image transformation with enhanced accuracy. Driven by the physical model of FBP, the dFBP network can be easily trained using small amounts of data yet is highly robust and versatile. The test results in animals and humans show that dFBP-based PAT can reconstruct high-quality artifact-suppressed images from sparse-view, limited-view, and acoustic heterogeneity-contaminated projection data and is well suited for PAT imaging under nonideal conditions. The proposed dFBP approach provides a practical solution for high-quality image recovery from incomplete projection data and can be used as a routine algorithm in PAT.

## Declaration of competing interest

The authors declare that they have no conflicts of interest in this work.

## Appendix Supplementary materials

Image, application 1
