# Supplementary material for: Physics-driven deep learning photoacoustic tomography
Source: Fundam Res. 2024 Sep 14;6(3):1715–26. doi: 10.1016/j.fmre.2024.06.014 (PMC13247502; doi:10.1016/j.fmre.2024.06.014)
Supplement: Supplementary file 1 [file mmc1.docx]

Supplementary materials for

Physics-driven deep learning photoacoustic tomography

Kang Shen^a,b,c^, Kuo Niu^c^, Songde Liu^a,c^, Yannis M. Paulus^d,e^, Xiaohua Jiang^f,*^, Chao Tian^a,b,c,g,*^

^a^Department of Anesthesia, The First Affiliated Hospital of USTC, Division of Life Sciences and Medicine, University of Science and Technology of China, Hefei, Anhui 230026, China

^b^Institute of Artificial Intelligence, Hefei Comprehensive National Science Center, School of Engineering Science, Hefei, Anhui 230088, China

^c^School of Engineering Science, University of Science and Technology of China, Hefei, Anhui 230026, China

^d^Department of Ophthalmology and Visual Sciences, University of Michigan, Ann Arbor, MI 48105, USA

^e^Department of Biomedical Engineering, University of Michigan, Ann Arbor, MI 48105, USA

^f^Center for Reproduction and Genetics, Department of Obstetrics and Gynecology, the First Affiliated Hospital of USTC, Division of Life Sciences and Medicine, University of Science and Technology of China, Hefei, Anhui 230026, China

^g^Anhui Province Key Laboratory of Biomedical Imaging and Intelligent Processing, Institute of Artificial Intelligence, Hefei Comprehensive National Science Center, Hefei 230088, China

* Corresponding author: [biojxh@ustc.edu.cn](mailto:biojxh@mail.ustc.edu.cn) (X. Jiang); [ctian@ustc.edu.cn](mailto:ctian@ustc.edu.cn) (C. Tian)

**The PDF file includes**:

**Fig. S1**. Signal detection and image reconstruction geometry in photoacoustic tomography (PAT).

**Fig. S2.** Principle of filtered back projection (FBP)-based image reconstruction.

**Fig. S3.** Schematic diagram of the custom-built PAT imaging system.

**Fig. S4**. Experimental study of the feasibility of the proposed dFBP algorithm.

**Fig. S5.** Difference images of a numerical zebrafish in the sparse-view PAT imaging experiment.

**Fig. S6**. dFBP-based PAT achieves high-quality imaging under sparse-view measurements for other slices of the numerical zebrafish.

**Fig. S7.** Difference images of an *in vivo* mouse in the sparse-view PAT imaging experiment.

**Fig. S8.** Difference images of a numerical zebrafish in the limited-view PAT imaging experiment.

**Fig. S9**. dFBP-based PAT achieves high-quality imaging under limited-view measurements for other slices of the numerical zebrafish.

**Fig. S10.** Difference images of an *in vivo* mouse in the limited-view PAT imaging experiment.

**Fig. S11.** Half-time-based image reconstruction fails to yield correct images in limited-view PAT imaging.

**Table S1.** Specifications of the proposed dFBP network.

**Table S2**. Summary of the size of the datasets used in this study.

**Note S1**: Comparison of different implementations of the ramp filtering subnetwork in the filtering module

**Note S2**: Comparison of different network architectures for the back-projection module

**Note S3**: Comparison of different normalization strategies

**Note S4**: Data preprocessing

**Note S5**: Evaluation metrics

**Note S6**: Comparative study of different deep learning-based PAT image reconstruction approaches

**Additional References**


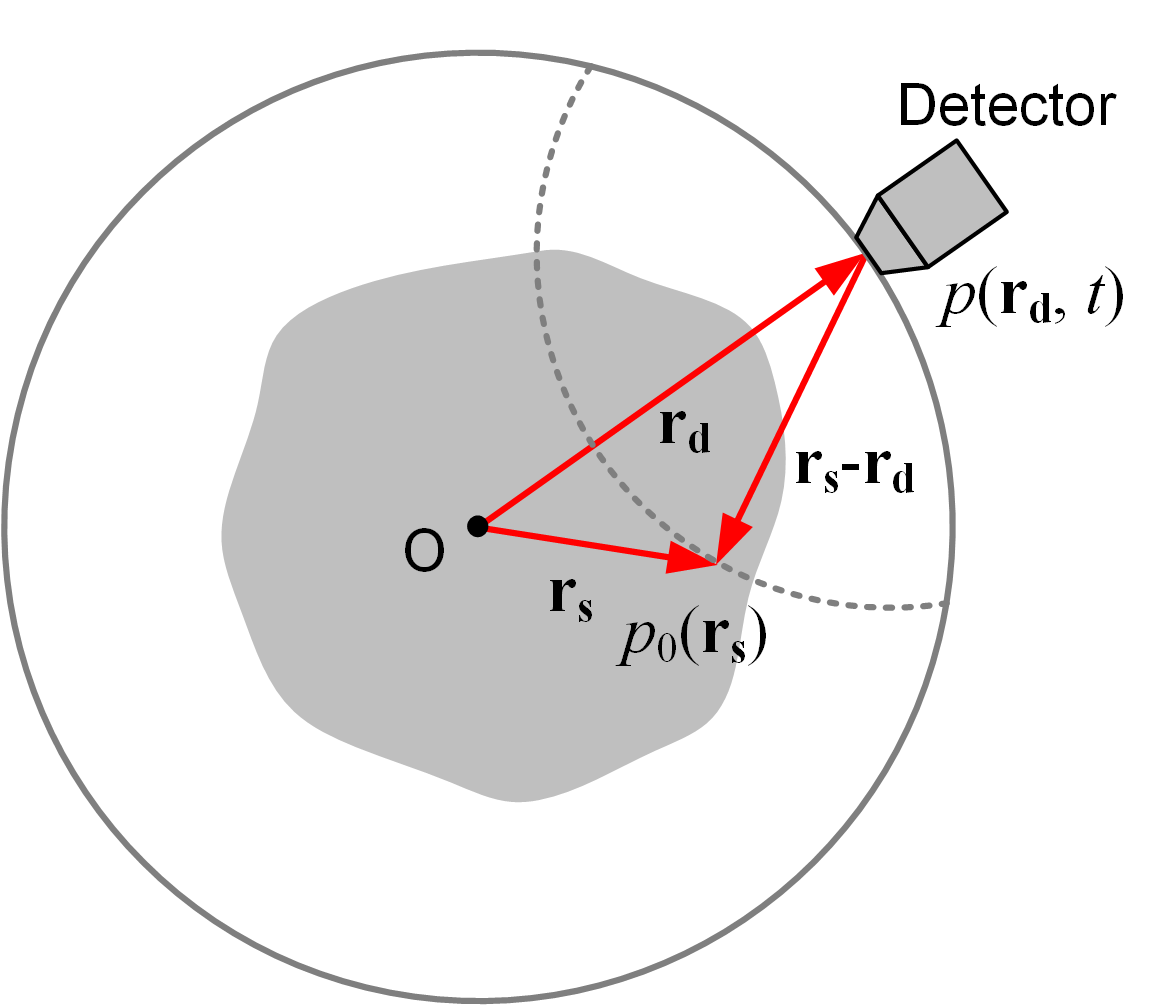


**Fig. S1.** **Signal detection and image reconstruction geometry in photoacoustic tomography (PAT).** **r**_d_: detector position; **r**_s_: source position.


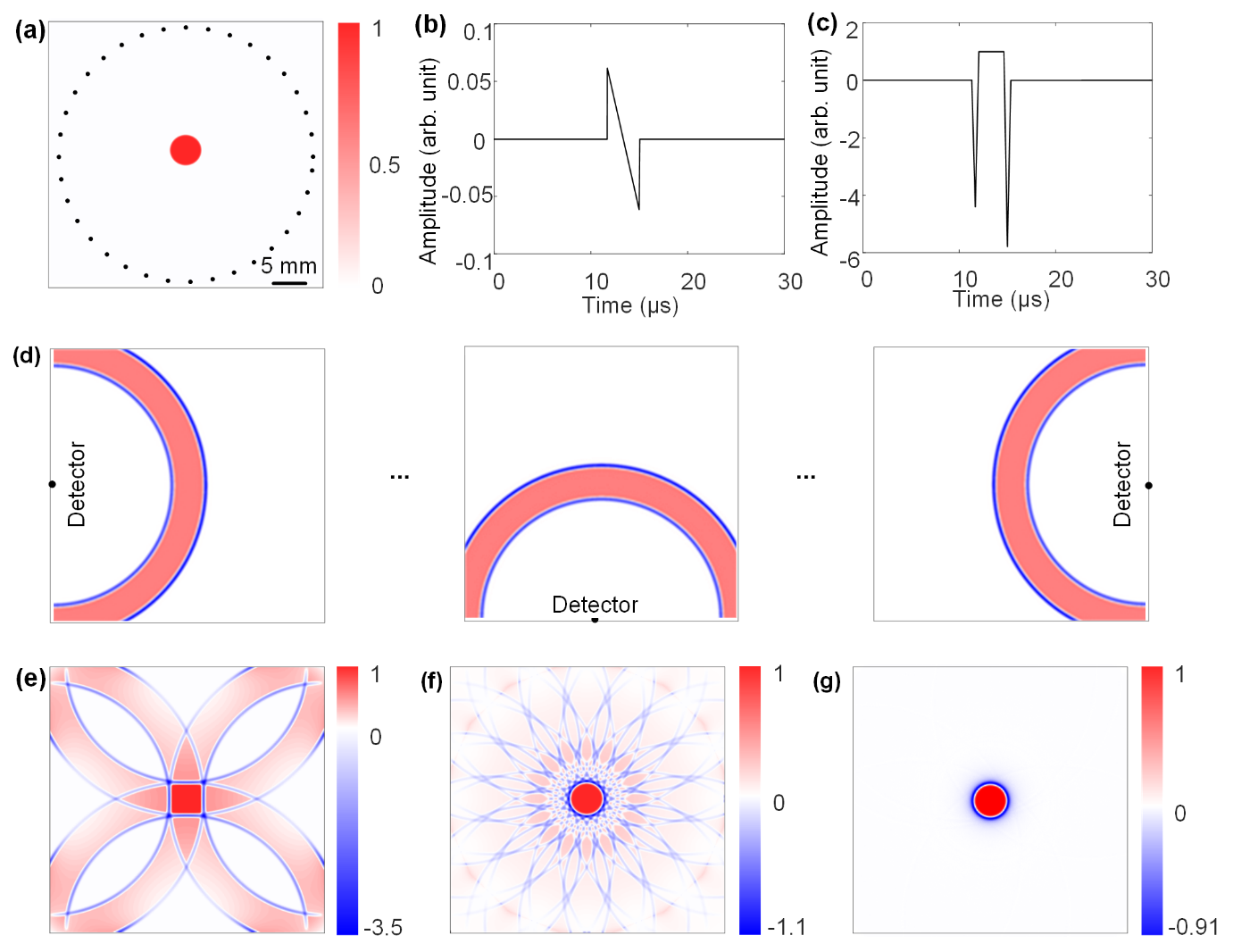


**Fig. S2. Principle of filtered back projection (FBP)-based image reconstruction.** (a) Schematic showing a spherical photoacoustic (PA) source (diameter: 5 mm) and an array of point detectors uniformly distributed over a circle (diameter: 40 mm). (b) Representative PA signal recorded by a detector on the measurement circle. (c) Back-projection signal [based on Eq. (5) in the main text]. (d) Projection images produced by detectors at different locations. (e)-(g) Final images formed by summing 4, 16, and 256 projection images, respectively.


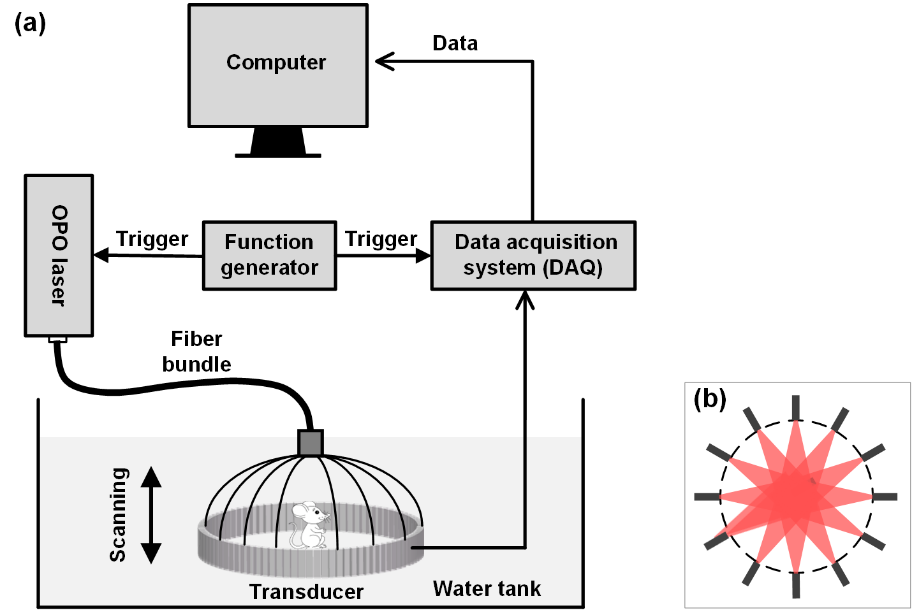


**Fig. S3. Schematic diagram of the custom-built PAT imaging system.** (a) Major components of the imaging system. (b) Ring-shaped laser illumination using 12 fiber bundles evenly distributed over a circle.


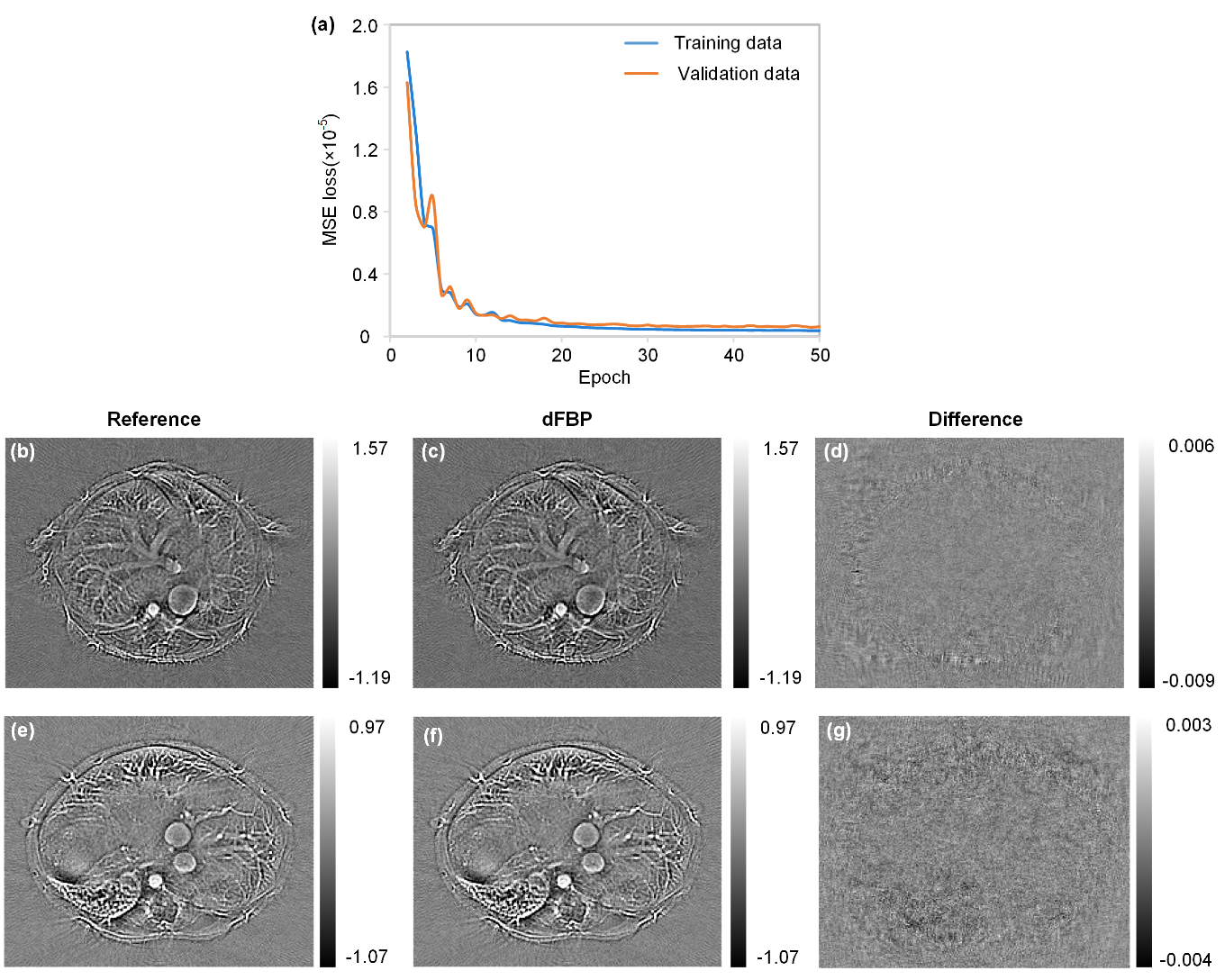


**Fig. S4.**  **Experimental study of the feasibility of the proposed dFBP algorithm.** (a) Loss curves in dFBP. Blue: training loss; orange: validation loss. (b) and (e) Two reference images reconstructed by FBP using 512-channel *in vivo* projection data acquired by the custom-built PAT imaging system. (c) and (f) Corresponding images reconstructed by dFBP. (d) and (g) Difference images between the reference images and the images reconstructed by dFBP. The results show that the proposed dFBP can achieve signal-to-image transformation on experimental dataset with high accuracy.


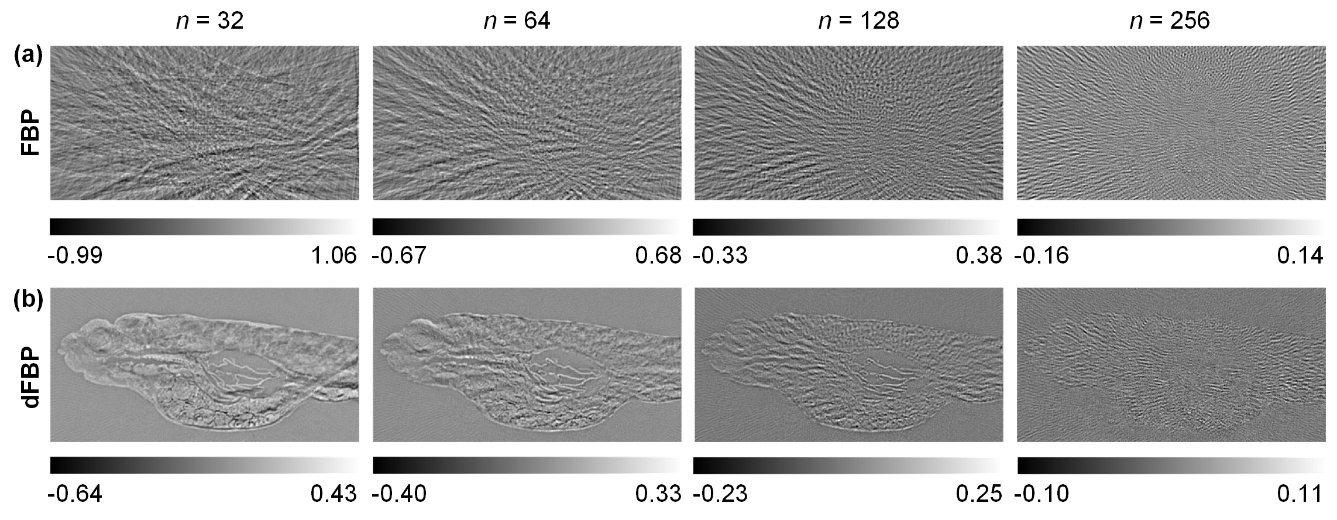


**Fig. S5.** **Difference images of a numerical zebrafish in the sparse-view PAT imaging experiment.** See the example in Fig. 3 in the main text for more details. (a) Difference images between the reference image and the FBP-reconstructed images under different numbers of views (from left to right: *n* = 32, 64, 128, and 256). (b) Difference images between the reference image and the dFBP-reconstructed images under different numbers of views (from left to right: *n* = 32, 64, 128, and 256). The results show that dFBP has smaller reconstruction errors than analytical FBP on the numerical zebrafish dataset in sparse-view imaging.


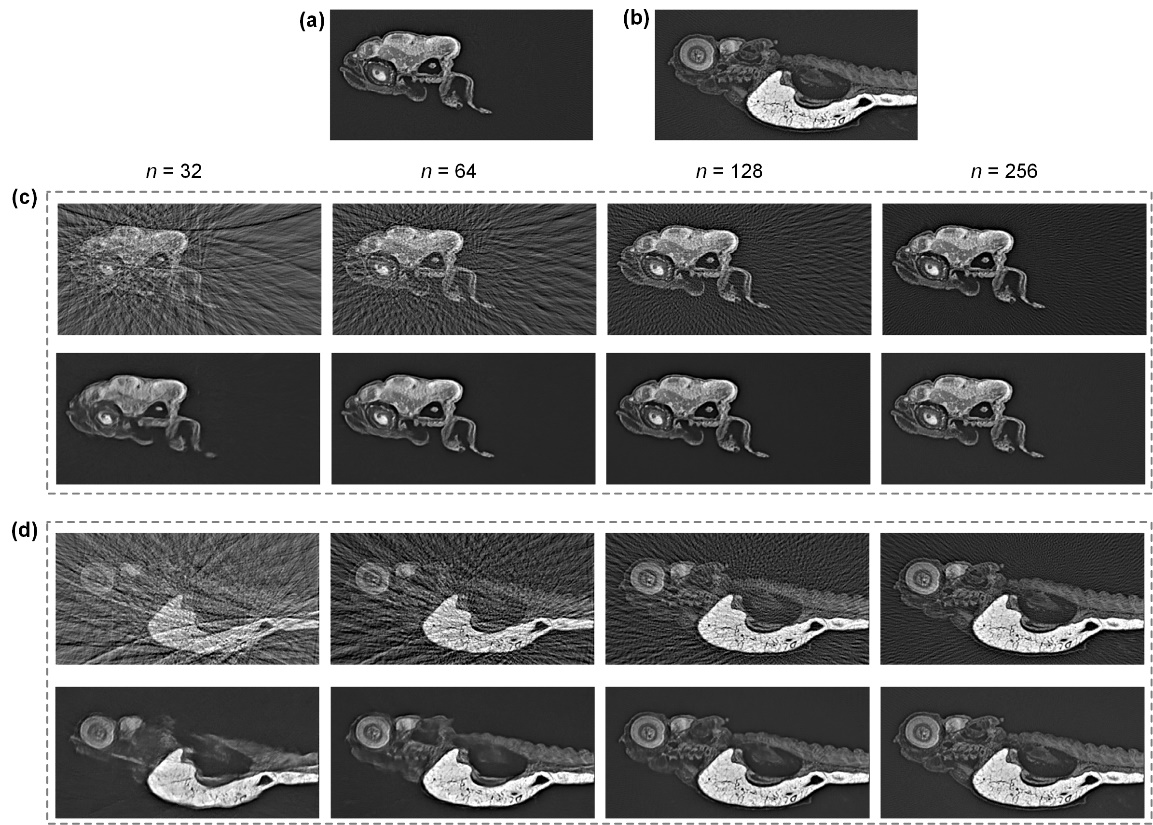


**Fig. S6.** **dFBP-based PAT achieves high-quality imaging under sparse-view measurements for other slices of the numerical zebrafish.** (a) and (b) Reference images of two other cross-sectional slices of the zebrafish. (c) and (d) Imaging results of the two slices under different numbers of views (from left to right: *n* = 32, 64, 128, and 256). The first and second rows are the results reconstructed by FBP and dFBP, respectively.


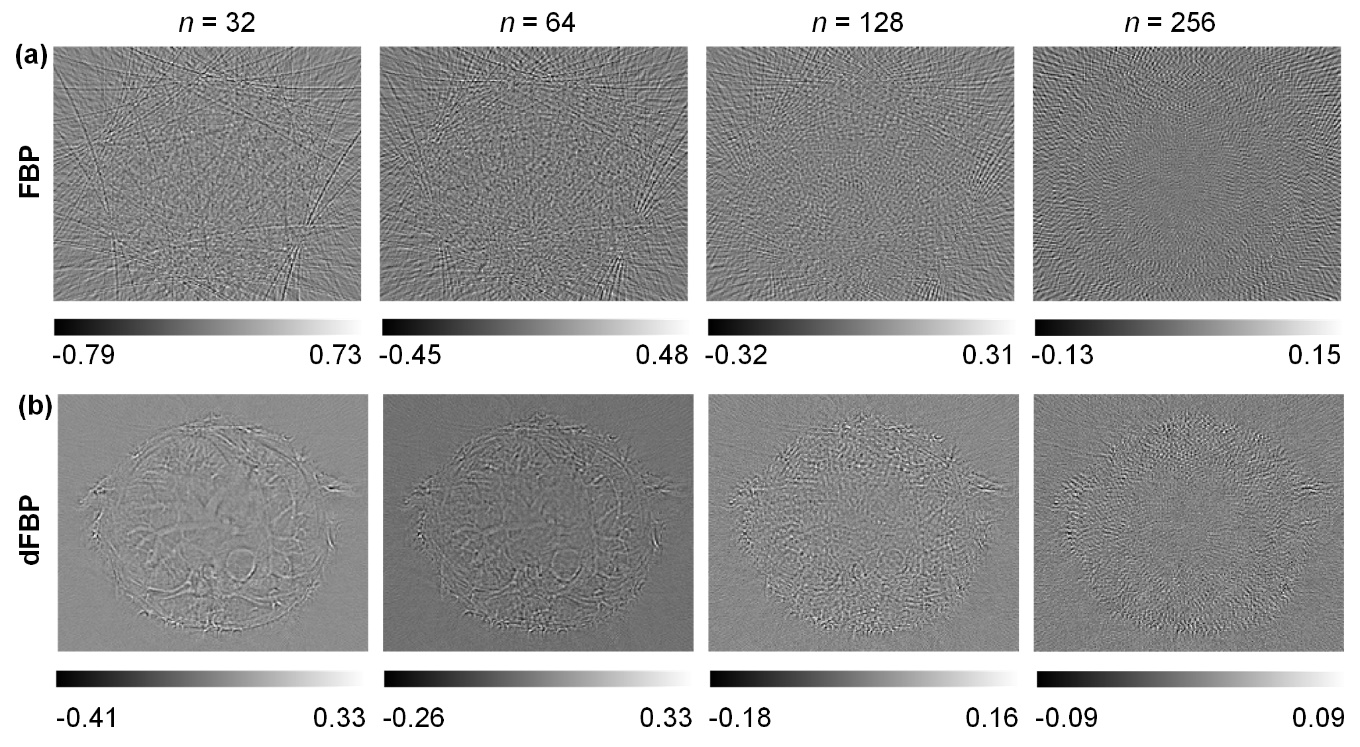


**Fig. S7.** **Difference images of an in vivo mouse in the sparse-view PAT imaging experiment.** See the example in Fig. 4 in the main text for more details. (a) Difference images between the reference image and the FBP-reconstructed images under different numbers of views (from left to right: *n* = 32, 64, 128, and 256). (b) Difference images between the reference image and the dFBP-reconstructed images under different numbers of views (from left to right: *n* = 32, 64, 128, and 256). The results show that dFBP has smaller reconstruction errors than analytical FBP on the in vivo mouse dataset in sparse-view imaging.


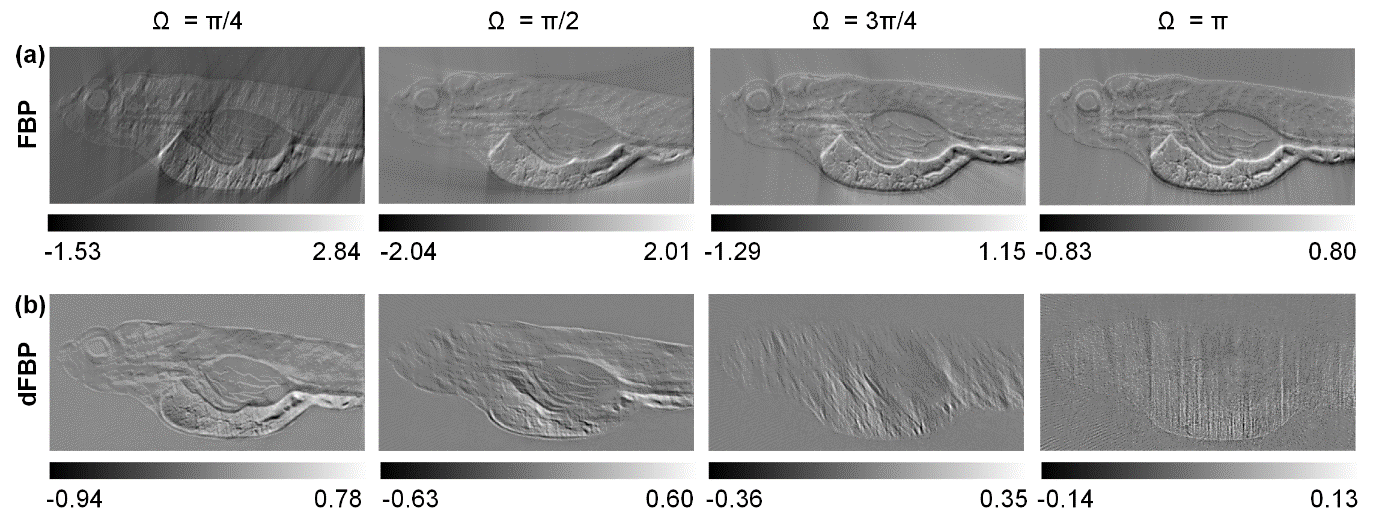


**Fig. S8.** **Difference images of a numerical zebrafish in the limited-view PAT imaging experiment.** See the example in Fig. 5 in the main text for more details. (a) Difference images between the reference image and the FBP-reconstructed images under different view angles (from left to right: Ω = π/4, π/2, 3π/4, and π). (b) Difference images between the reference image and the dFBP-reconstructed images under different view angles (from left to right: Ω = π/4, π/2, 3π/4, and π). The results show that dFBP has smaller reconstruction errors than analytical FBP on the numerical zebrafish dataset in limited-view imaging.


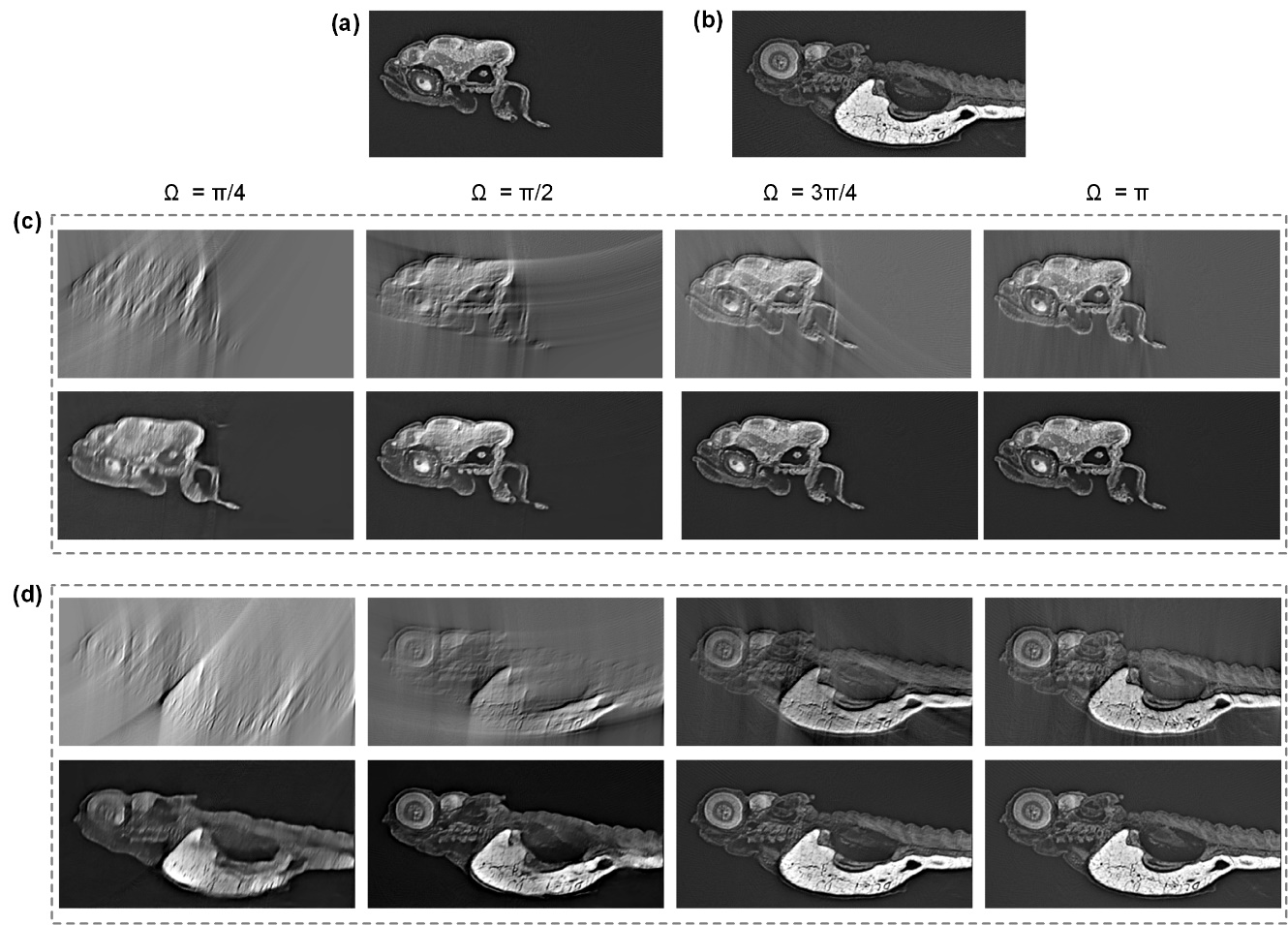


**Fig. S9.** **dFBP-based PAT achieves high-quality imaging under limited-view measurements for other slices of the numerical zebrafish.** (a) and (b) Reference images of two other cross-sectional slices of the zebrafish. (c) and (d) Imaging results of the two slices under different view angles (from left to right: Ω = π/4, π/2, 3π/4, and π). The first and second rows are the results reconstructed by FBP and dFBP, respectively.


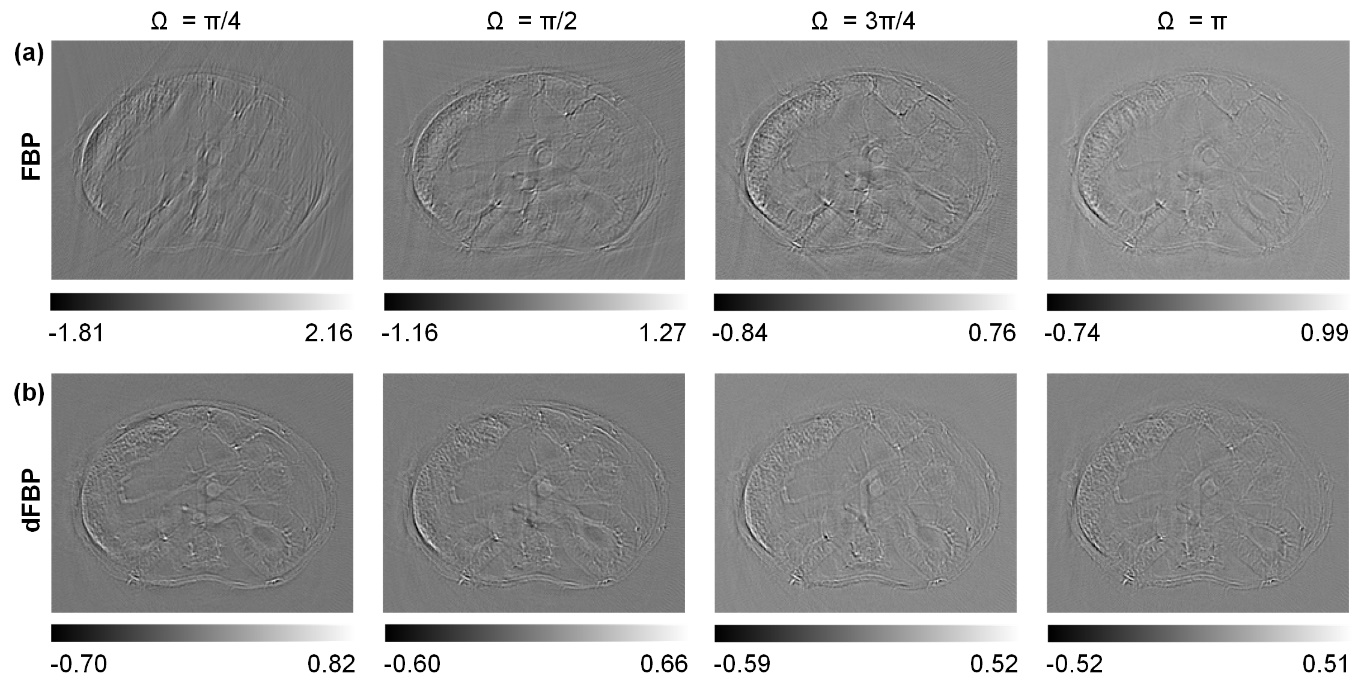


**Fig. S10.** **Difference images of an in vivo mouse in the limited-view PAT imaging experiment.** See the example in Fig. 8 in the main text for more details. (a) Difference images between the reference image and the FBP-reconstructed images under different view angles (from left to right: Ω = π/4, π/2, 3π/4, and π). (b) Difference images between the reference image and the dFBP-reconstructed images under different view angles (from left to right: Ω = π/4, π/2, 3π/4, and π). The results show that dFBP has smaller reconstruction errors than analytical FBP on the in vivo mouse dataset in limited-view imaging.


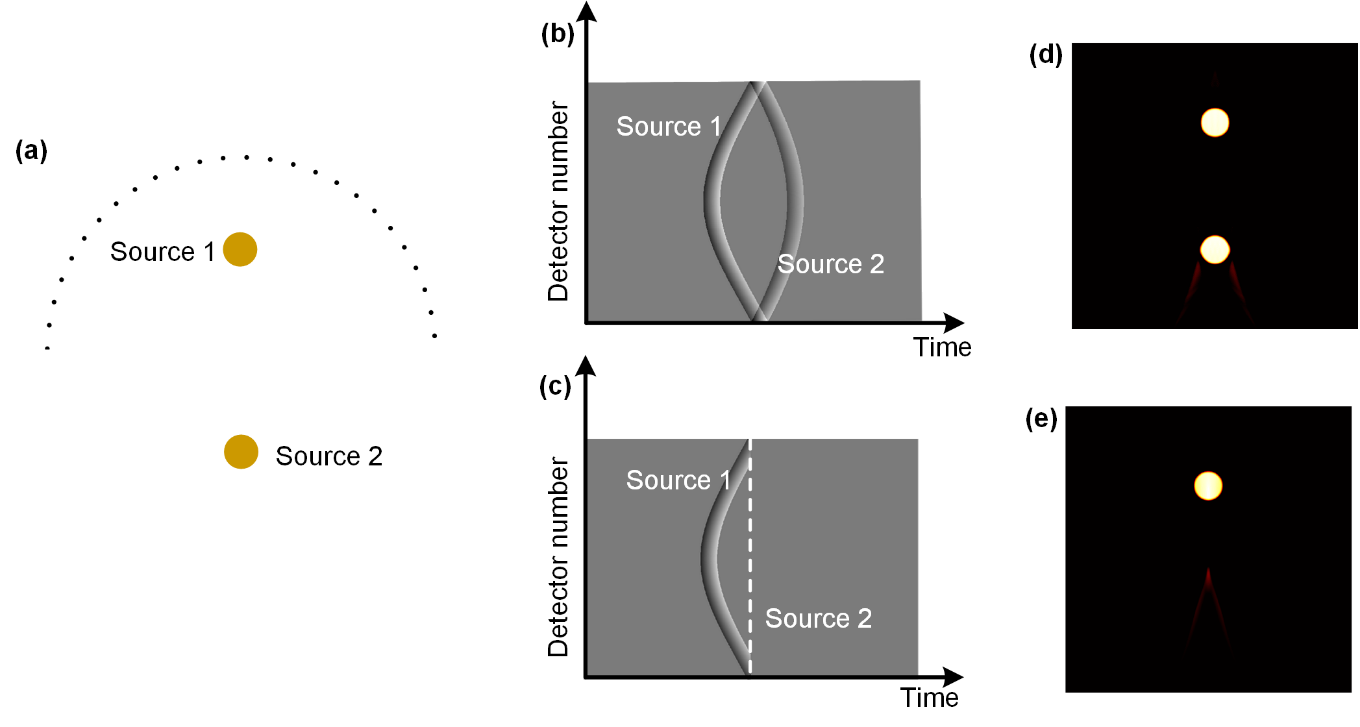


**Fig. S11.** **Half-time-based image reconstruction fails to yield correct images in limited-view PAT imaging.** (a) Schematic showing a limited-view imaging scenario, where two disc-shaped PA sources (diameter: 4 mm) are imaged by a half-ring detector array (diameter: 40 mm, detector number: 256, view angle Ω: π). (b) Full-time projection data of the PA sources recorded by the detector array. (c) Half-time projection data. The dashed line represents the cut-off time in the half-time approach. (d) Image reconstructed by FBP using the full-time projection data in (b). (e) Image reconstructed by FBP using the half-time projection data in (c). The image produced by the half-time approach in this case is incorrect.

**Table S1. Specifications of the proposed dFBP network.**

| **Layer (L)** | | **Operation** | **Filter** | **Output**  **[H, W, C]** | **Connected from** |
| --- | --- | --- | --- | --- | --- |
| **Input** | 1 | - | - | [*N_d_*, *N_s_*, 1] | - |
| **Filtering**  **module** | 2 | Conv (1 × 189), Tanh | 16 | [*N_d_*, *N_s_*, 16] | L1 |
|  | 3 | Conv (1 × 189), Tanh | 1 | [*N_d_*, *N_s_*, 1] | L2 |
|  | 4 | Permute (1, 3, 2) | - | [*N_d_*, 1, *N_s_*] | L3 |
|  | 5 | Conv (1 × 1) | *N*_s_ | [*N_d_*, 1, *N_s_*] | L4 |
|  | 6 | Permute (1, 3, 2) | - | [*N_d_*, *N_s_*, 1] | L5 |
|  | 7 | Matrix multiply (·) | 1 | [*N_d_*, *N_s_*, 1] | L6 |
|  | 8 | Subtract (·) | - | [*N_d_*, *N_s_*, 1] | L1, 7 |
| **Back-projection**  **module** | 9 | Reshape (1, *N_d_* × *N*_s_) | - | [1, *N_d_* × *N*_s_, -] | L8 |
|  | 10 | Transpose (·) | - | [*N_d_* × *N_s_*, 1, -] | L9 |
|  | 11 | Sparse matrix matmul (·) | 1 | [*N_x_* × *N_y_*, 1, -] | L10 |
|  | 12 | Transpose (·) | - | [1, *N_x_* × *N_y_*, -] | L11 |
|  | 13 | Reshape (*N_x_*, *N_y_*, 1) | - | [*N_x_*, *N_y_*, 1] | L12 |
|  | 14 | Tile (1, 1, *N_d_*) | - | [*N_x_*, *N_y_*, *N_d_*] | L13 |
|  | 15 | Matrix Multiply (·) | 1 | [*N_x_*, *N_y_*, *N_d_*] | L14 |
| **Fusion module** | 16 | Conv (3 × 3), GN, ReLU | 64 | [*N_x_*, *N_y_*, 64] | L15 |
|  | Encoder (contractive path) | | | | |
|  | 17 | 2 × [Conv (3 × 3), GN, ReLU] | 64 | [*N_x_*, *N_y_*, 64] | L16 |
|  | 18 | Pooling (2 × 2) | - | [*N_x_*/2, *N_y_*/2, 64] | L17 |
|  | 19 | 2 × [Conv (3 × 3), GN, ReLU] | 128 | [*N_x_*/2, *N_y_*/2, 128] | L18 |
|  | 20 | Pooling (2 × 2) | - | [*N_x_*/4, *N_y_*/4, 128] | L19 |
|  | 21 | 2 × [Conv (3 × 3), GN, ReLU] | 256 | [*N_x_*/4, *N_y_*/4, 256] | L20 |
|  | 22 | Pooling (2 × 2) | - | [*N_x_*/8, *N_y_*/8, 256] | L21 |
|  | 23 | 2 × [Conv (3 × 3), GN, ReLU] | 512 | [*N_x_*/8, *N_y_*/8, 512] | L22 |
|  | 24 | Pooling (2 × 2) | - | [*N_x_*/16, *N_y_*/16, 512] | L23 |
|  | 25 | 2 × [Conv (3 × 3), GN, ReLU] | 1024 | [*N_x_*/16, *N_y_*/16, 1024] | L24 |
|  | Decoder (expansive path) | | | | |
|  | 26 | Pooling (8 × 8),  Conv (3 × 3), GN, ReLU | 64 | [*N_x_*/8, *N_y_*/8, 64] | L16 |
|  | 27 | Pooling (4 × 4),  Conv (3 × 3), GN, ReLU | 64 | [*N_x_*/8, *N_y_*/8, 64] | L18 |
|  | 28 | Pooling (2 × 2),  Conv (3 × 3), GN, ReLU | 64 | [*N_x_*/8, *N_y_*/8, 64] | L20 |
|  | 29 | Conv (3 × 3), GN, ReLU | 64 | [*N_x_*/8, *N_y_*/8, 64] | L22 |
|  | 30 | Upsampling (2 × 2),  Conv (3 × 3), GN, ReLU | 64 | [*N_x_*/8, *N_y_*/8, 64] | L25 |
|  | 31 | Concatenate (·),  Conv (3 × 3), GN, ReLU | - | [*N_x_*/8, *N_y_*/8, 320] | L26, 27, 28, 29, 30 |
|  | 32 | Pooling (4 × 4),  Conv (3 × 3), GN, ReLU | 64 | [*N_x_*/4, *N_y_*/4, 64] | L16 |
|  | 33 | Pooling (2 × 2),  Conv (3 × 3), GN, ReLU | 64 | [*N_x_*/4, *N_y_*/4, 64] | L18 |
|  | 34 | Conv (3 × 3), GN, ReLU | 64 | [*N_x_*/4, *N_y_*/4, 64] | L20 |
|  | 35 | Upsampling (2 × 2),  Conv (3 × 3), GN, ReLU | 64 | [*N_x_*/4, *N*_y_/4, 64] | L31 |
|  | 36 | Upsampling (4 × 4),  Conv (3 × 3), GN, ReLU | 64 | [*N_x_*/4, *N_y_*/4, 64] | L25 |
|  | 37 | Concatenate (·),  Conv (3 × 3), GN, ReLU | - | [*N_x_*/4, *N_y_*/4, 320] | L32, 33, 34, 35, 36 |
|  | 38 | Pooling (2 × 2),  Conv (3 × 3), GN, ReLU | 64 | [*N_x_*/2, *N_y_*/2, 64] | L16 |
|  | 39 | Conv (3 × 3), GN, ReLU | 64 | [*N_x_*/2, *N_y_*/2, 64] | L20 |
|  | 40 | Upsampling (2 × 2),  Conv (3 × 3), GN, ReLU | 64 | [*N_x_*/2, *N_y_*/2, 64] | L37 |
|  | 41 | Upsampling (4 × 4),  Conv (3 × 3), GN, ReLU | 64 | [*N_x_*/2, *N_y_*/2, 64] | L31 |
|  | 42 | Upsampling (8 × 8),  Conv (3 × 3), GN, ReLU | 64 | [*N*_x_/2, *N*_y_/2, 64] | L25 |
|  | 43 | Concatenate (·),  Conv (3 × 3), GN, ReLU | - | [*N_x_*/2, *N_y_*/2, 320] | L37, 38, 39, 40, 41 |
|  | 44 | Conv (3 × 3), GN, ReLU | 64 | [*N_x_*, *N_y_*, 64] | L16 |
|  | 45 | Upsampling (2 × 2),  Conv (3 × 3), GN, ReLU | 64 | [*N_x_,* *N_y_*, 64] | L43 |
|  | 46 | Upsampling (4 × 4),  Conv (3 × 3), GN, ReLU | 64 | [*N*_x_, *N*_y_, 64] | L37 |
|  | 47 | Upsampling (8 × 8),  Conv (3 × 3), GN, ReLU | 64 | [*N_x_*, *N_y_*, 64] | L31 |
|  | 48 | Upsampling (16 × 16),  Conv (3 × 3), GN, ReLU | 64 | [*N_x_,* *N_y_*, 64] | L25 |
|  | 49 | Concatenate (·),  Conv (3 × 3), GN, ReLU | - | [*N_x_*, *N_y_*, 320] | L44, 45, 46, 47, 48 |
|  | 50 | Conv (3 × 3) | 1 | [*N_x_*, *N_y_*, 1] | L49 |
|  | 51 | Conv (1 × 1) | 1 | [*N_x_*, *N_y_*, 1] | L15 |
| **Output** | 52 | Add (·) | - | [*N_x_*, *N_y_*, 1] | L50, 51 |

**Table S2. Summary of the size of the datasets used in this study.**

| **Datasets** | **Size of PA signals** | **Size of PA images** | **Number of volumes** | **Number of slices** |
| --- | --- | --- | --- | --- |
| Numerical mouse embryo (I) | 800 × *N_d1_* | 192 × 192 | 51 | 2878 |
| Numerical mouse embryo (II) | 800 × *N_d1_* | 192 × 192 | 1 | 10 |
| Numerical zebrafish | 750 × *N_d2_* | 192 × 384 | 5 | 4448 |
| *In vivo* mouse | 750 × *N_d2_* | 256 × 256 | 15 | 3062 |
| *In vivo* human finger | 750 × *N_d2_* | 256 × 256 | 10 | 963 |

*N_d1_*: number of detectors on the spherical measurement geometry (*N_d1_* = 2048).

*N_d2_*: number of detectors on the partially or fully circular measurement geometry (*N_d2_* = 32 to 512).

**Note S1: Comparison of different implementations of the ramp filtering subnetwork in the filtering module**

The implementation of ramp filtering of input PA signals is critical to the success of the filtering module in dFBP. In this study, the ramp filtering subnetwork was constructed based on three convolutional layers, each of which is followed by an activation function [[1](#_ENREF_1)]. The ReLU activation function (Fig. S12a) is a common activation function used in neural networks, which outputs zero for negative input and outputs the input directly otherwise. However, since PA signals are bipolar and contain both positive and negative components, the nonnegative ReLU activation function may not work best for the ramp filtering subnetwork. In contrast, the Tanh activation function (Fig. S12b) allows both the negative and positive components of PA signals to pass and is preferable in this study. In addition, a linear activation function (Fig. S12c) that outputs an equal value to the input is usually included in the output layer or regression layer of the ramp filtering subnetwork to achieve enhanced fitting. Figure S12d-g shows the architectures of four ramp filtering networks with different activation function combinations. Figure S13 illustrates an example showing the practical performance of the four networks for the filtering of raw PA projection data. The results show that the network with two Tanh activation functions followed by a linear activation function (Fig. S12g) has the best performance.


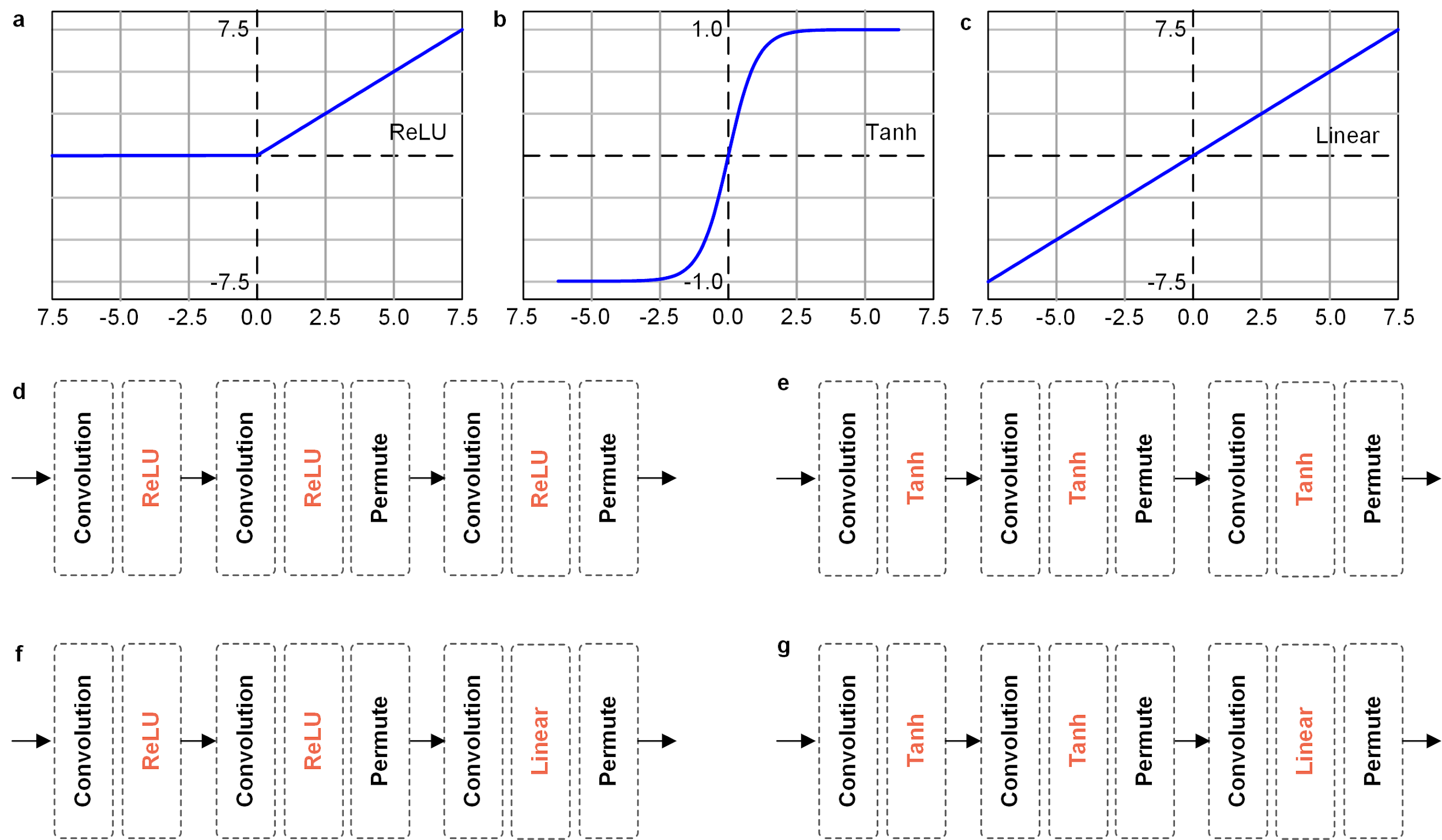


**Fig. S12.** **Different implementations of the ramp filtering subnetwork in the filtering module.** (a)-(c) Graphical diagrams of the ReLU, Tanh, and linear activation functions. (d)-(g) Four ramp filtering subnetworks with different activation function combinations. (d) ReLU + ReLU + ReLU. (e) Tanh + Tanh + Tanh. (f) ReLU + ReLU + Linear. (g) Tanh + Tanh + Linear.


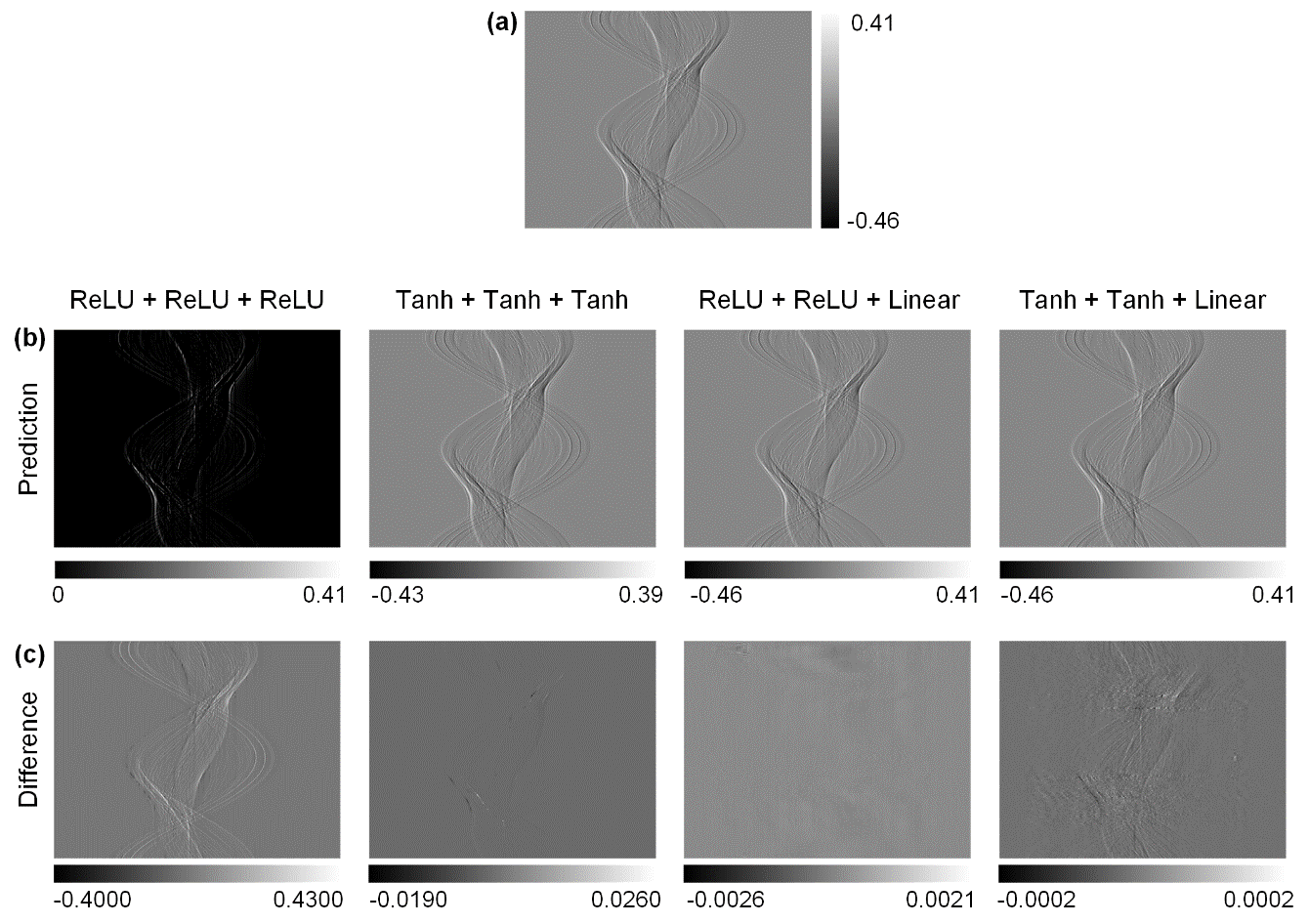


**Fig. S13.** **An example showing the performance of the four ramp filtering subnetworks.** (a) Theoretical filtered PA signals for reference. (b) Filtering results output by the four ramp filtering subnetworks. (c) Differences between the reference and the corresponding predicted filtering data. The results show that two Tanh activation functions followed by a linear activation function yield the best results.

**Note S2: Comparison o****f different network architectures for the back-projection module**

The back-projection module aims to transform the back-projection terms output by the filtering module into a series of projection images. To achieve such a transformation, one of the most straightforward approaches is to use fully connected layers, as shown in Fig. S14a, where the back-projection terms corresponding to detectors at different positions are mapped into a series of projection images by multiple fully connected layers. However, the use of multiple fully connected layers involves a large number of parameters to be trained (*N_d_N_s_* × *N_x_N_y_*, where *N_s_* is the number of sampling points of PA signals for each detector, *N_d_* is the number of detectors, and *N_x_* and *N_y_* are the sizes of the image being reconstructed) and imposes huge demands on training data and computing hardware. Moreover, the fully-connected-layers based approach can easily lead to overfitting and increase the difficulty for network training from the data domain to the image domain. Noticing that the back-projection transformations for different detectors are identical except for the spatial position of each detector, it is possible to deploy a single fully connected layer plus a spatial coordinate mapping layer to achieve back projection for all detectors, as shown in Fig. S14b. This can dramatically reduce the number of parameters to be trained from *N_d_N_s_* × *N_x_N_y_* to *N_s_* × *N_x_N_y_*. However, the extra-introduced spatial coordinate transformation varies from geometry to geometry and may cause misalignment errors. To address these problems, we designed an efficient transformation layer based on a transformation matrix (TM) and a decomposition matrix (DM) as shown in Fig. S14c. The TM transforms the back-projection terms of all detectors into a low-quality 2D image, and the DM decomposes the low-quality PA image into a series of 2D projection images, each corresponding to the PA signal of a detector. Please refer to Section 3.1 of the main text for detailed descriptions of the TM and DM. This approach reduces the total number of parameters to be trained to *N_s_* × *N_x_N_y_*, the same as the second approach but does not involve any spatial coordinate transformation. Table S3 shows the comparison of the three network architectures in terms of the number of involved parameters and the requirement on the shape of measurement geometry.


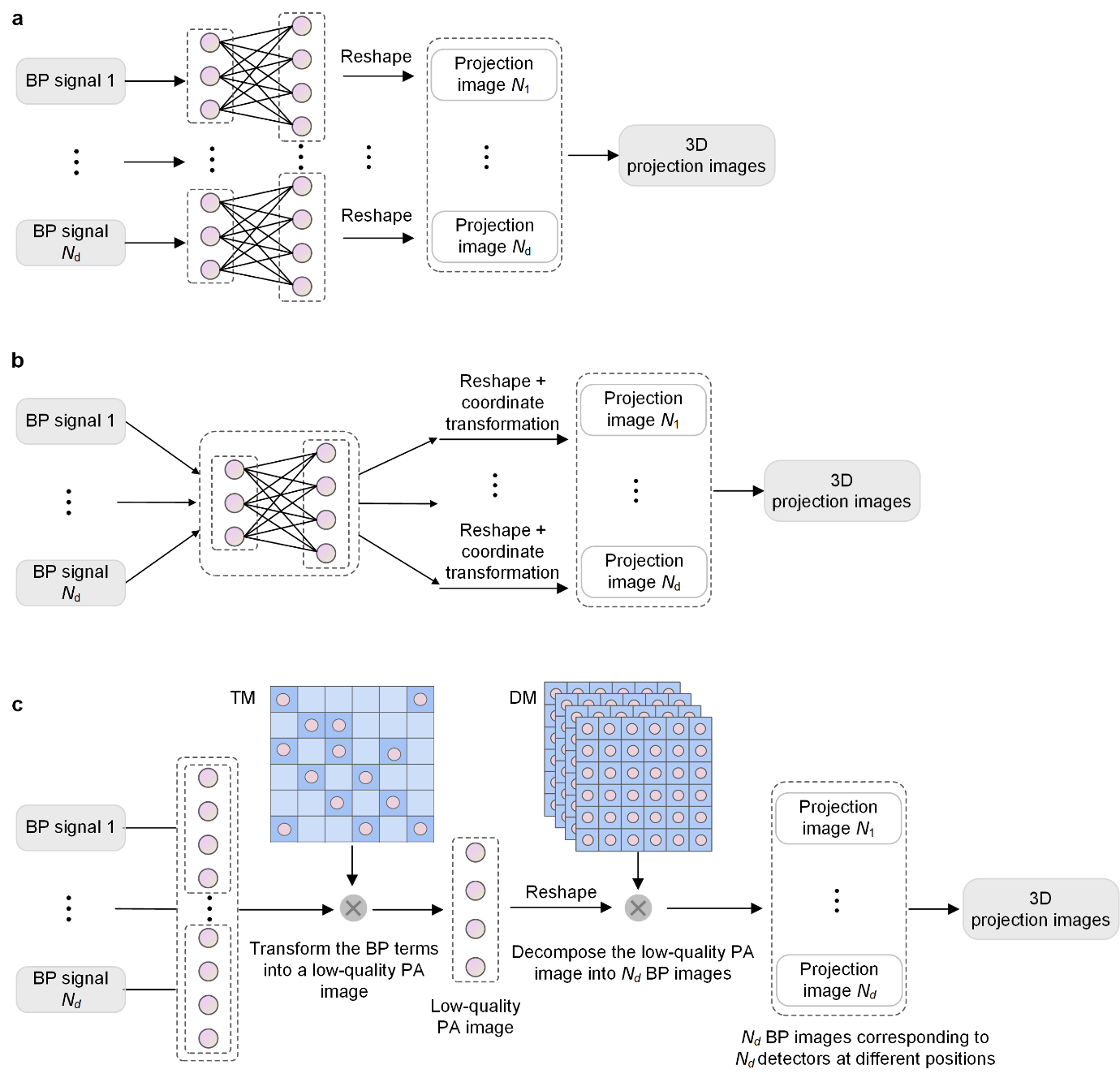


**Fig. S14.** **Comparison of different network implementations of the back-projection (BP) module.** (a) BP module based on multiple fully connected layers. (b) BP module based on a fully connected layer and a coordinate transformation layer. (c) BP module based on a TM and a 3D DM. The input of the module is the back-projection terms output by the filtering module, which have a size of 1 × *N_d_N_s_*. The output of the module is 3D projection images with a size of *N_x_* × *N_y_* × *N_d_*. *N_d_* is the number of detectors, *N_s_* is the number of sampling points of PA signals of each detector, *N_x_* and *N_y_* are the sizes of the image being reconstructed.

**Table S3. Comparison of different network architectures for the back-projection module in terms of the number of involved parameters and the requirement on the measurement geometry.**

| **Network architecture** | **Number of parameters** | **Requirement on the measurement geometry** |
| --- | --- | --- |
| Multiple fully connected layers | *N_d_N_s_* × *N_x_N_y_* | Arbitrary |
| One fully connected layer  + one coordinate mapping layer | *N_s_* × *N_x_N_y_* | Specific |
| A sparse transformation matrix +  a decomposition matrix | *N_d_* × *N_x_N_y_* | Arbitrary |

**Note S3: Comparison of different normalization strategies**

The normalization layer in deep learning neural networks can accelerate the training of the network and improve prediction accuracy. One well-known normalization method is batch normalization [[2](#_ENREF_2)], which uses statistical metrics such as the mean and variance to describe features within a batch. The batch normalization can be mathematically expressed as

$\hat{x}_{i}=\frac{1}{\sigma_{i}}\left(x_{i}-\mu_{i}\right),$ (S1)

where *x_i_* and $\hat{x}_{i}$ are the input feature and the normalized feature, respectively. The index *i* = (*i_N_*, *i_C_*, *i_H_*, *i_W_*) is used to index a feature from the input, where *N* is the batch size, *C* is the channel number, and *H* and *W* are the height and width of the image, respectively (Fig. S15a). *μ_i_* and *σ_i_* in Eq. (S8) denote the mean and the standard deviation of the features, respectively, and can be calculated as

$\mu_{i}=\frac{1}{m}\sum_{k\in S_{i}}^{m} x_{k},$ (S2)

$\sigma_{i}=\sqrt{\frac{1}{m}\sum_{k\in S_{i}} \left(x_{k}-\mu_{i}\right)^{2}+\varepsilon},$ (S3)

where *ε* is a small positive constant to avoid *σ_i_* = 0 and the set *S_i_* is defined as

$S_{i}=\left\{k|k_{C}=i_{C}\right\}.$ (S4)

Here *i_C_* and *k_C_* represent the subindices of *i* and *k* along the channel axis, respectively. In batch normalization, the elements having the same channel index are normalized together. However, the batch normalization method generally requires a large batch size. Otherwise, it may give inaccurate estimations.

To address this problem, a group normalization strategy [[3](#_ENREF_3)], as shown in Fig. S15b, can be used. Group normalization is similar to batch normalization except for the definition of *S_i_*. In group normalization, the set *S_i_* is defined as

$S_{i}=\left\{k|k_{N}=i_{N},\left\lfloor\frac{k_{C}}{C/G}\right\rfloor=\left\lfloor\frac{i_{C}}{C/G}\right\rfloor\right\},$ (S5)

where *G* is the number of groups and equals 16 in this work; and the symbol represents the floor operation. $\left\lfloor\frac{k_{C}}{C/G}\right\rfloor=\left\lfloor\frac{i_{C}}{C/G}\right\rfloor$ indicates that the indices *k* and *i* are in the same group, where C/G is the group size. Group normalization performs better when the batch size is small.


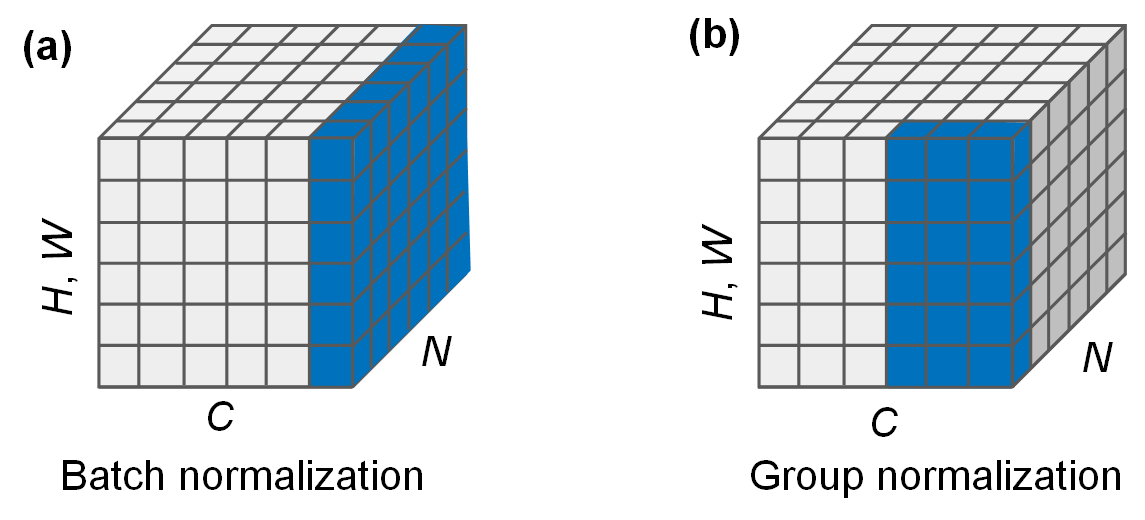


**Fig. S15.** (a) Batch normalization. (b) Group normalization. *N* is the batch size, *C* is the channel number, and *H* and *W* are the height and width of the image, respectively. The elements in blue are normalized together.

Note S4: Data preprocessing

Although the imaging region in the custom-built PAT system can be the whole area enclosed by the full-ring transducer array, the ROI is typically around the center of the transducer. Therefore, it is reasonable to discard irrelevant projection data and only retain the essential portion that reflects the information of the ROI. This can improve computational efficiency while preserving useful information. To achieve this, we only keep raw PA projection data that satisfy the simple condition

$d_{\min}\text{/}v\leq t\leq d_{\max}/v,$ (S6)

where *d*_min_ and *d*_max_ are the minimum and maximum distances from the detector to the ROI, respectively. Figure S16 illustrates the basic idea of the data preprocessing strategy.


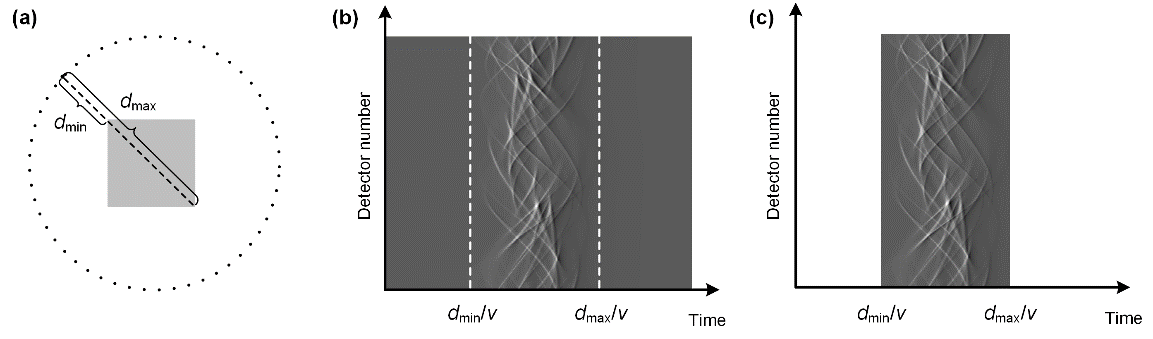


**Fig. S16.** **Preprocessing strategy for measured PA projection data.** (a) Schematic showing the relative position of a circular detector array and the effective imaging region indicated by the square in gray. dmin and dmax denote the minimum and maximum distances between the detector array and the reconstruction region, respectively. (b) PA projection data recorded by the circular detector array. The dashed lines indicate the boundary of useful data. *v*: sound speed. (c) Retained data for image reconstruction.

**Note S5: Evaluation metrics**

To quantitatively evaluate the performance of different methods, three popular metrics, including RMSE, PSNR, and SSIM [[4](#_ENREF_4)], are used to measure the quality of reconstructed images. The RMSE is defined as

$\text{RMSE}=\left[\frac{1}{M\times N}\left\|x-x'\right\|_{2}^{2}\right]^{\text{1/2}},$ (S7)

where *x* and *x*ʹ are the reference image and the reconstructed image, respectively, and *M* and *N* are image sizes. The PSNR is defined as

$\text{PSNR}=10\log_{10}\frac{\left[\max(x)\right]^{2}}{\text{MSE}},$ (S8)

where max(*x*) denotes the maximum value of the image *x* and MSE represents the mean square error. The SSIM is defined as

$\text{SSIM}=\frac{(2\mu_{x}\mu_{x'}+C_{1})(2\sigma_{xx'}+C_{2})}{(\mu_{x}^{2}+\mu_{x'}^{2}+C_{1})(\sigma_{x}^{2}+\sigma_{x'}^{2}+C_{2})},$ (S9)

where $\mu_{x}$ and $\mu_{x'}$ are the mean of the reference image *x* and the reconstructed image *x*ʹ, respectively; $\sigma_{x}$ and $\sigma_{x'}$ are the variance of the reference image *x* and the reconstructed image *x*ʹ, respectively; $\sigma_{xx'}$ represents the covariance between the reference image *x* and the reconstructed image *x*ʹ; $C_{1}=(0.01\max(x))^{2}$; and $C_{2}=(0.03\max(x))^{2}$.

**Note S6: Comparative study of different deep learning-based PAT image reconstruction approaches**

To further evaluate the performance of the proposed dFBP network, we conducted a comparative study to compare the performance of dFBP with two other state-of-the-art deep learning-based PAT image reconstruction methods under sparse sampling. The first is an end-to-end UNet with residual blocks (Res-UNet) proposed by Feng and coworkers [[5](#_ENREF_5)] and the second is a feature projection network (FPnet) plus a UNet proposed by Tong and colleagues [[6](#_ENREF_6)]. The two approaches can both achieve signal-to-image reconstruction in sparse-view and limited-view PAT imaging. However, they are prone to overfitting and require a large amount of training data to learn the mapping function between PA projection data and PA images. Therefore, they may not perform well for ill-posed image reconstruction in experimental settings.

To illustrate this point, we carried out the comparative study using the *in vivo* mouse dataset, which contains 512-channel whole-body PA projection data of 15 different living mice (see the Methods section in the main text for the details of the dataset). The comparison conditions are set as follows. In the end-to-end Res-UNet and the FPnet-UNet experiment, 13 mice were used for network training, 2 mice were used for validation, and 1 mouse from the training set was used for testing. In the proposed dFBP, 12 mice are used for network training, 2 mice were used for validation, and 1 mouse independent from the training set was used for testing. Apparently, the training and test conditions are more demanding for the proposed dFBP method. Figure 17a-c shows the reconstruction results of a representative PA image using 128-channel projection data by the end-to-end Res-UNet, FPNet-UNet, and dFBP, respectively. Figure 17d is the reference image reconstructed using 512-channel projection data by FBP. The results show that distortions and blurring are present in the images reconstructed by the end-to-end Res-UNet and FPNet-UNet. In contrast, dFBP suppresses artifacts, recovers more structures, and produces images with the highest quality. Figure 17e-h shows another example, where dFBP similarly gives the best result, illustrating the robustness of the proposed method. To quantitatively evaluate the performance of the three methods, the RMSE, PSNR, and SSIM of the reconstructed images were calculated based on the whole test dataset and are shown in Figure 17i-k. The images reconstructed by dFBP have the lowest RMSE value and the highest PSNR and SSIM values, which confirms the effectiveness of the proposed method.


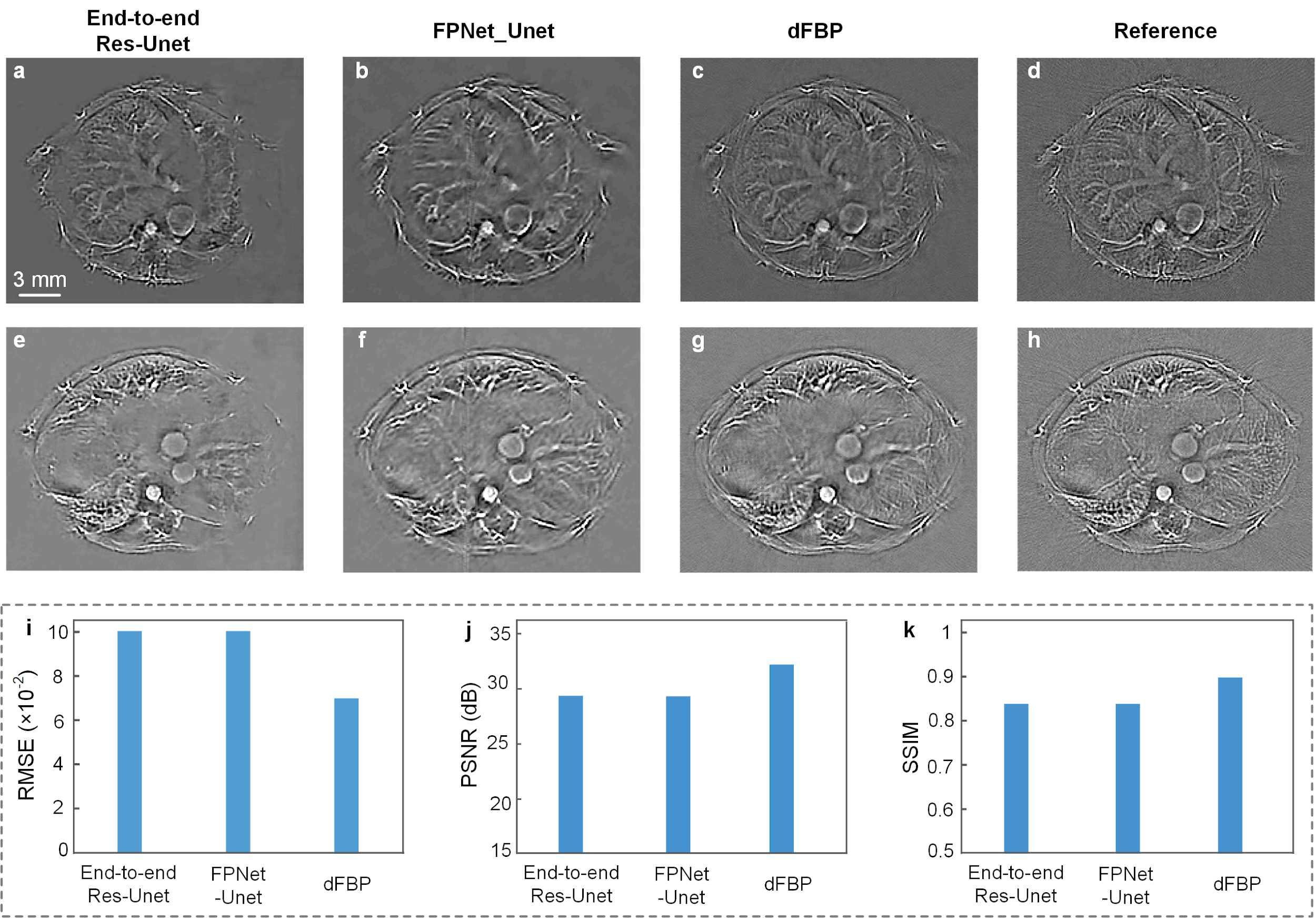


**Fig. S17. Performance comparison of different deep learning-based PAT image reconstruction methods based on *in vivo* mouse data.** (a) and (e) Two representative PA images reconstructed by the end-to-end Res-UNet [[5](#_ENREF_5)] using 128-channel projection data. (b) and (f) PA images reconstructed by FPNet-UNet [[6](#_ENREF_6)] using 128-channel projection data. (c) and (g) PA images reconstructed by the proposed dFBP using 128-channel projection data. (d) and (h) Corresponding reference images reconstructed by FBP using 512-channel projection data. (i)-(k) Quantitative evaluation of the images reconstructed by the three methods. The results show that the proposed dFBP is substantially superior to the other two methods.

**Additional References**

[1] Glorot X, Bordes A, Bengio Y. Deep sparse rectifier neural networks[J]. In Proceedings of the 14th International Conference on Artificial Intelligence and Statistics, 2011, 15: 315-323.

[2] Ioffe S, Szegedy C. Batch normalization: Accelerating deep network training by reducing internal covariate shift[J]. In Proceedings of Machine Learning Research, 2015, 37: 448-456.

[3] Wu Y, He K. Group normalization[J]. In Proceedings of the European Conference on Computer Vision, 2018: 3-19.

[4] Wang Z, Bovik A C, Sheikh H R, et al. Image quality assessment: From error visibility to structural similarity[J]. IEEE Trans Image Process, 2004, 13(4): 600-612.

[5] Feng J, Deng J, Li Z, et al. End-to-end Res-Unet based reconstruction algorithm for photoacoustic imaging[J]. Biomed Opt Express, 2020, 11(9): 5321-5340.

[6] Tong T, Huang W, Wang K, et al. Domain transform network for photoacoustic tomography from limited-view and sparsely sampled data[J]. Photoacoustics, 2020, 19(2020): 100190.
